# The Potential Hepatocyte Differentiation Targets and MSC Proliferation by FH1

**DOI:** 10.1111/jcmm.70601

**Published:** 2025-05-10

**Authors:** Sang Luo, Fang Wu, Yiran Jin, Dan Liu

**Affiliations:** ^1^ Department of Beijing National Biochip Research Center Sub‐Center in Ningxia, Institute of Medical Sciences General Hospital of Ningxia Medical University Yinchuan China; ^2^ Ningxia Regional Key Laboratory of Integrated Traditional Chinese and Western Medicine for Prevention and Treatment of Regional High Incidence Disease Yinchuan China; ^3^ Key Laboratory of Ministry of Education for Fertility Preservation and Maintenance Ningxia Medical University Yinchuan China; ^4^ Department of Gynecology General Hospital of Ningxia Medical University Yinchuan China

**Keywords:** acute liver failure, functional hit 1, hepatocyte differentiation, HGF/c‐met signalling, mesenchymal stem/stromal cells

## Abstract

The main cause of acute liver failure (ALF) is hepatocellular necrosis, which induces liver repair dysfunction and leads to high mortality. In recent years, studies have increasingly shown that stem cell‐derived hepatocyte‐like cells (HLCs) can be used for treatment in animal models of ALF. Notably, a hepatocyte differentiation strategy based on the small‐molecule compound functional hit 1 (FH1) successfully replaces HGF to promote the maturation of HLCs, but the underlying mechanism is still unclear. In this study, we used network pharmacology analysis to clarify the important role of the HGF/c‐Met signalling pathway in FH1‐induced hepatocyte (FH1‐iHeps) differentiation. After FH1 was added to mesenchymal stem/stromal cells (MSCs), proliferation and cell cycle progression were rescued by treatment with a tyrosine kinase (c‐Met) inhibitor. Additionally, c‐Met signalling in MSCs was significantly increased by treatment with FH1, as shown by the increased c‐Met, p‐p38, p‐AKT and p‐ERK1/2 protein levels. FH1‐iHeps efficiently improved the liver function of mice with acute liver injury and prolonged their lifespan. These data provide new insight into the mechanisms regulating the stemness properties of human umbilical cord‐derived stem cells (hUC‐MSCs) and reveal a previously unrecognised link between FH1 and c‐Met in directing hepatocyte differentiation.

**Abbreviations**
ALB: albumin
ALF: acute liver failure
CCl_4_
: carbon tetrachloride
FH1: functional hit 1
HB: hepatoblasts
HS: hepatic specification
hUC‐MSCs: human umbilical cord mesenchymal stem/stromal cells

## 1 Introduction

Acute liver failure (ALF) is a major clinical issue and economic burden worldwide due to the high mortality rate caused by hepatocellular necrosis [1, 2, 3]. To date, orthotopic liver transplantation (OLT) is an effective treatment for ALF; however, due to the shortage of available donor livers, financial burden and requirement for surgery, many patients die while waiting for liver transplantation [4, 5].

Since Dr. Groth's successfully conducted mouse hepatocyte transplantation in 1977 [6], the first successful human hepatocyte transplantation in humans was not reported until 1992, when Dr. Mito in Japan and Drs. Strom and Fisher in the United States. described autologous transplants and allogeneic transplants, respectively [7]. Nonetheless, the application of hepatocyte transplantation has many restrictions because these cells easily lose their hepatocytic functions and cannot be passaged in vitro [8]. Therefore, identifying new cell lines to meet the requirement for high‐quality hepatocytes is urgently needed. In recent years, studies have increasingly shown that mesenchymal stem/stromal cells (MSCs) are an ideal source of seed cells for treating ALF [9].

To date, researchers have developed multiple stem cell types, namely, embryonic stem cells, induced pluripotent stem cells, MSCs, hepatic stem cells [9], amniotic epithelial stem cells [10] and liver progenitor cells [11], for use in hepatic differentiation. MSCs are the most common type of cells used for hepatic differentiation because they are easy to obtain and have no ethical restrictions as well as easy to expand ex vivo and can be transplanted allogenically without the need of immunosuppression to increase tolerance to the graft [12]. The traditional method [13, 14, 15, 16, 17] for inducing MSCs to differentiate into hepatocytes is time‐consuming and high cost of cytokines; in our previous work, we successfully established a protocol based on the small molecule functional hit 1 (FH1) for the differentiation of MSCs into hepatocyte‐like cells (HLCs) without using HGF within only 11 days [18].

FH1 is a small molecule compound with a molecular weight of 282.34 Da that was discovered in a study conducted by Harvard Medical School in 2013; the study focused on compounds related to liver cell proliferation [19]. This compound belongs to a family of compounds that promote liver cell functional maturation and is therefore named FH1. Du et al. found that FH1 can induce the directed differentiation of pluripotent stem cell‐derived liver progenitor cells into mature liver cells and increase the expression levels of albumin (ALB), alpha fetoprotein and cytochrome enzymes in induced liver cells while promoting the morphological formation of liver cells [20]. However, the mechanism controlling FH1‐driven hepatic differentiation remains unclear.

The maturation of hepatocytes involves the participation of multiple signalling pathways. Greenbaum and Wells [21] reported that the expansion and directional differentiation of hepatic progenitor cells depend on signals provided by tissue‐specific stem cell niches. Among these signals, mesenchymal‐derived hepatocyte growth factor (HGF) can bind to its cognate receptor tyrosine kinase Met, thereby inducing and activating downstream signalling molecules such as extracellular signal‐regulated kinase 1/2 (ERK1/2), p38, MAPK, STAT3 and PI3K/Akt [22]. In the mature stage of hepatocyte development, HGF/c‐Met signalling serves as an effective mitogen for hepatocytes and is crucial for the differentiation of hepatic progenitor cells [23]. Wang et al. [24] demonstrated that HGF‐mediated liver regeneration is related to the high expression of c‐Met in hepatocytes. These discoveries strongly suggested that HGF/c‐Met is a crucial driver involved in hepatic differentiation. However, the relationship between FH1 and HGF/c‐Met is still unclear. Hence, in this study, we aimed to explore the effect of FH1 on MSC proliferation and identify an association between FH1 and hepatic differentiation induced by c‐Met signalling.

## 2 Materials and Methods

### 2.1 Network Pharmacology Analysis

#### 2.1.1 Identification of the Target of FH1

The SMILES structure of FH1 was acquired from the PubChem database (https://pubchem.ncbi.nlm.nih.gov/), and the targets of FH1 were searched with the search bar of the SEA database (http://sea.bkslab.org). In the PharmMapper database (http://lilab‐ecust.cn/pharmmapper/), the predicted target of FH1 was obtained by searching for the keyword ‘Functional hit 1’. The union of the predicted targets for the compound obtained from the three databases was the final predicted target of FH1.

#### 2.1.2 Prediction of Hepatocyte Maturation/Differentiation Targets

The targets for hepatocyte maturation/differentiation were obtained from the OMIM database (https://omim.org/) and GeneCards (human gene information database http://www.genecards.org/). The search results from these databases were integrated, and any duplicated targets were eliminated.

#### 2.1.3 Target Interaction Network

To compare liver differentiation/maturation and FH1 targets, we used the Kidio Bio website (https://www.omicshare.com/) to create a Venn diagram and screen out common targets. The common targets were entered into the String database (http://string‐db.org/), the species was limited to *Homo sapiens* and the target interaction network was obtained.

#### 2.1.4 Enrichment Analysis of Common Targets

The Metascape database (http://metascape.org/gp/index.html#/main/step1) was used to obtain the annotation information of Gene Ontology (GO) functional enrichment analysis, and the annotated information was used to perform GO (biological processes, molecular functions, cellular components) analysis and Kyoto Encyclopedia of Genes and Genomes (KEGG) pathway enrichment analysis of the intersecting genes.

#### 2.1.5 Gene Set Enrichment Analysis

Gene set enrichment analysis (GSEA) is supported by the broad institute website (http://www.broadinstitute.org/gsea/index.jsp) and includes versions compatible with Java, R, or Gene Pattern. GSEA was performed to determine whether a priori defined set of genes shows statistically significant results in biological processes and molecular functions.

#### 2.1.6 Molecular Docking

The 3D structural files of FH1 in SDF were obtained from the PubChem database. The target protein structural files were downloaded from UniProt. Finally, molecular docking simulations were conducted using the CB‐Dock2 database (https://cadd.labshare.cn/cb‐dock2).

#### 2.1.7 BLI Analysis

FH1 (Abmole Bioscience) and the target protein (Sino Biological) were prepared, and BLI analysis was performed according to previous methods [25]. The target protein was biotinylated in vitro using a biotinylation kit (GENEMORE) according to the manufacturer's instructions. A Super Streptavidin Biosensor (SSA) was purchased from Sartorius, and the SSA biosensor was prewetted with PBST (0.02% Tween 20). Biotinylated target proteins in 96‐well black F‐bottom plates were directly immobilised on the SSA sensor. FH1 was diluted to an appropriate concentration with PBST containing 15% DMSO for a final volume of 200 μL per well. Then, an equal volume of 15% DMSO in PBST was added to the wells as a blank control. The cycle repetition parameters were set, namely, baseline 60 s, loading 600 s, baseline 60 s, binding 60 s, dissociation 60 s and five other steps. The data were analysed using Sartorius Octet data acquisition and data analysis software.

### 2.2 Cell Experiments

#### 2.2.1 Cell Culture

The culture method for human umbilical cord‐derived stem cells (hUC‐MSCs) was established by our laboratory [18, 26] (RC02003, Nuwacell Biotechnologies Co. Ltd., Hefei, China). Briefly, hUC‐MSCs at passage 3 were cultured in serum‐free human MSC (SF‐hMSC) basal medium supplemented with supplement (RP020101, Nuwacell ncMission hMSC Basal Medium; RP02010‐2, Nuwacell ncMission Supplement, Nuwacell Biotechnologies Co. Ltd.). Then, the cells were cultured at 37°C in a humidified atmosphere in a 5% CO_2_ incubator for 3–4 days until the confluence reached 85%.

#### 2.2.2 Hepatic Differentiation of hUC‐MSCs In Vitro

In vitro hepatic differentiation of hUC‐MSCs was performed as described previously [18, 26]. The primary small molecules used in the definitive endoderm differentiation stage were all‐trans retinoic acid (ATRA, 1 μM; Abmole Bioscience), IDE1 (100 nM), CHIR99021 (3 μM; Abmole Bioscience) and LY294002 (10 μM; Abmole Bioscience). In the progenitor hepatic formation stage, the cells were incubated in serum‐free IMDM supplemented with 100 nM IDE1 (MCE), 10 μM LY294002, 250 nM LDN‐193189 (Abmole Bioscience) and 20 ng/mL basic fibroblast growth factor (bFGF; PeproTech) for 48 h, after which the medium was changed to serum‐free IMDM supplemented with 100 nM IDE1, 10 μM LY294002 and 20 ng/mL bFGF for another 24 h. For the hepatocyte maturation stage, the maturation medium was composed of William's E medium (Gibco) supplemented with 15 μM FH1 (Abmole Bioscience), 20 ng/mL bFGF, 30 ng/mL oncostatin M (OSM; PeproTech), 2 × 10^−5^ mol/L dexamethasone (Dex; Abmole Bioscience) and 1% insulin‐transferrin‐selenium supplement‐X (ITS‐X; Sigma Aldrich). The cells were cultured in maturation medium for 5 days, after which the medium was changed every 48 h.

#### 2.2.3 Actin Staining

For fluorescence staining of the actin cytoskeleton, the cells were fixed with Immunol staining fix solution (Beyotime) for 20 min. After fixation, the cells were washed three times with PBS containing 0.1% Triton X‐100 for 5 min and stained with Actin‐Tracker Green‐488 (Beyotime) for 60 min in a dark room. After three washes with PBS containing 0.1% Triton X‐100 for 5 min, the cells were visualised by fluorescence microscopy (Olympus). Each experiment was repeated three independent times.

#### 2.2.4 EdU Analysis

At the end of each treatment, the medium of the hUC‐MSCs was changed to form 5‐ethynyl‐2′‐deoxyuridine (EdU; Beyotime Biotechnology, Shanghai, China). The reagents were diluted with culture medium (to a final concentration of 20 μM) and incubated for 2 h. The cells were then harvested and fixed with 4% paraformaldehyde for 15 min at room temperature and rinsed with PBS for 3 min. The cells were then blocked with 0.3% Triton X‐100 (dissolved in PBS) for 15 min at room temperature. Next, 0.5 mL of click reaction reagent was added to each sample, which was then left for 30 min in a dark room. After the cells were rinsed 3 times with PBS, their proliferation was analysed via flow cytometry, and the results were visualised via fluorescence microscopy (Olympus). Each experiment was repeated three independent times.

#### 2.2.5 Transwell Migration Assay

Transwell assays were used to determine the migratory abilities of the cells. First, 200 μL of medium containing 2 × 10^5^ cells was seeded on the upper side of a porous polycarbonate membrane (pore size: 8 μm). Five hundred microliters of medium with or without FH1 were added to the lower compartment. After incubation at 37°C in 5% CO_2_ for 48 h, the cells were preincubated with PF4217903 (Abmole) for 1 h for inhibition experiments. At the end of the incubation, the cells on the upper side of the filter were mechanically removed. Cells that had migrated to the lower side of the filter were fixed with 11% glutaraldehyde for 30 min and stained with haematoxylin and eosin (H&E). Each experiment was repeated three independent times.

#### 2.2.6 Cell Cycle Analysis

At the end of each treatment, the hUC‐MSCs were harvested, and a Cell Cycle Analysis Kit (no. C1052; Beyotime, Shanghai, China) was used to conduct the cell cycle assay. Briefly, hUC‐MSCs were fixed in 70% ethanol for 2 h at 4°C and then stained with 1× iodide staining solution containing 0.05 mg/mL propidium iodide, 1 mg/mL RNase A, and 0.3% Triton X‐100. The cells were incubated in the dark for 30 min. The percentage of cells in different phases of the cell cycle was examined via a flow cytometer (Beckman Coulter, Brea, CA) by measuring the DNA content (propidium iodide intensity). The quantitation of the cell cycle distribution was performed using ModFIT software. The percentages of G1‐, S‐ and G2/M‐phase cells were calculated. Each experiment was repeated three independent times.

#### 2.2.7 Western Blotting

Western blotting was performed as previously described [16, 24]. The primary antibodies used in this study were c‐Met (1:500; Proteintech; 25869‐1‐AP), phospho‐Met (1:1000; CST; 3077), β‐catenin (1:1000; Proteintech; 66379‐1‐lg), phospho‐β‐catenin (1:1000; CST; 4176), ERK (1:1000; Proteintech; 1125‐1‐AP), phospho‐ERK1/2 (1:1000; Proteintech; 28733‐1‐AP), AKT (1:1000; Proteintech; 60203‐2‐lg), phospho‐AKT (1:1000; Proteintech; 8‐455‐1‐RR), P38 (1:1000; Proteintech; 14064‐1‐AP), phospho‐P38 (1:1000; Proteintech; 2896‐1‐AP), P27 (1:1000; Proteintech; 25614‐1‐AP), P21 (1:1000; Proteintech; 10355‐1‐AP), BCL‐2 (1:1000; Sevicebio; GB124830) and PCNA (1:1000; Sevicebio; GB11010‐1). Goat anti‐rabbit IgG‐horseradish peroxidase (HRP) secondary antibody (1:5000; Sevicebio; GB23303) or goat anti‐mouse IgG‐HRP secondary antibody (1:5000; Sevicebio; GB23301) was used as the secondary antibody. Human actin was used as a control for protein loading (1:5000; Sevicebio; GB15003).

#### 2.2.8 RNA Sequencing

Total RNA was extracted from hUC‐MSCs, FH1‐treated hUC‐MSCs and FH1‐derived induced HLCs. After quality control of the RNA amount, purity and integrity, 200–500 bp cDNA library products from 2 μg of total RNA were quantified and finally sequenced on a DNBSEQ‐T7 sequencer (MGI Tech Co. Ltd., China) with the PE150 model. Differentially expressed genes were defined as those with a fold change > 2 or < 0.5 and *p* < 0.05, and then GO analysis and KEGG enrichment analysis were both implemented with KOBAS software (version 2.1.1). All the services used were provided by Seqhealth Technology Co. Ltd. (Wuhan, China).

#### 2.2.9 Data Download

The datasets of primary hepatocytes (GEO: GSM3683651, GSM3683652), fetal hepatocytes (FL, GEO: GSM6598973, GSM6598974), maturation hepatocytes (MH, GEO: GSM6598965, GSM6598969) [27, 28], hepatic specification (HS, GEO: GSM3683641; GSM3683642) and hepatoblasts (HB, GEO: GSM3683643, GSM3683644) were acquired from the NCBI GEO public database.

### 2.3 Animal Experiments

#### 2.3.1 Laboratory Animals

A total of 18 C57BL/6J mice (weighing 16–18 g, aged 6–8 weeks) were purchased from Spiff Biotechnology Company and maintained on 12 h light/dark cycles with food and water available at the Laboratory of Ningxia Medical University. All animals were treated in accordance with the principles of the Declaration of Helsinki and ARRIVE guidelines. The animal procedure described here was reviewed and approved by the Ethics Committee of General Hospital of Ningxia Medical University (NO. KYLL‐2023‐0169).

#### 2.3.2 Inclusion and Exclusion Criteria

The animals were included in the study if they successfully received carbon tetrachloride (CCl_4_) intraperitoneal injection, defined by inflammatory cells infiltrating the stroma through haematoxylin–eosin staining. The animals were excluded if they received no cell therapy and were also alive for 72 h, preventing the collection of behavioural and histological data.

#### 2.3.3 Blinding/Masking

For each animal, four different researchers participated in conducting the experiment. The first investigator was responsible for the anaesthetic procedure. The second investigator administered the treatment according to the randomisation table. The third investigator performed the CCl_4_ intraperitoneal injection. Finally, the fourth investigator carried out cell transplantation.

#### 2.3.4 Experimental Grouping and Intervention Method

The animals were randomly divided into three groups (*n* = 6/group): a control group, a hUC‐MSC transplantation group, and an induced hepatocyte transplantation group. C57BL/6J mice in the transplantation groups were injected with CCl_4_ olive oil solution (1:1)at a dose of 5 mL/kg body weight through intraperitoneal injection. After treatment with CCl_4_ for 8 h, the animals were injected with hUC‐MSCs or FH1‐induced hepatocytes (FH1‐iHeps) (1 × 10^6^, 300 μL) by intravenous injection. The control animals were treated with an equal volume of PBS.

#### 2.3.5 Histopathology

After 7 days of hUC‐MSCs or FH1‐iHeps infusion, the mice were euthanised by pentobarbital, and liver tissues were isolated and fixed in 10% paraformaldehyde. The fixed livers were then embedded in paraffin for H&E and periodic acid‐Schiff (PAS) (Beijing Solarbio Science & Technology Co. Ltd.) staining according to the instructions.

#### 2.3.6 Immunofluorescence Assays

Immunofluorescence assays were performed as previously described [16, 24]. The primary antibody used in this study was against ALB (16475‐1‐AP, Proteintech, 1:200) and the secondary antibody was CY3 goat anti‐rabbit IgG (H + L) (AS007, ABclonal, 1:200).

#### 2.3.7 Statistical Analysis

All the data are presented as the mean ± SD. Statistical analysis was performed with GraphPad Prism 6.0.1 software by two‐tailed unpaired Student's *t* tests and one‐way or two‐way ANOVA.

## 3 Results

### 3.1 Network Pharmacology Analysis

#### 3.1.1 Identification of the Target of FH1 in Hepatocyte Differentiation

**FIGURE 1:**
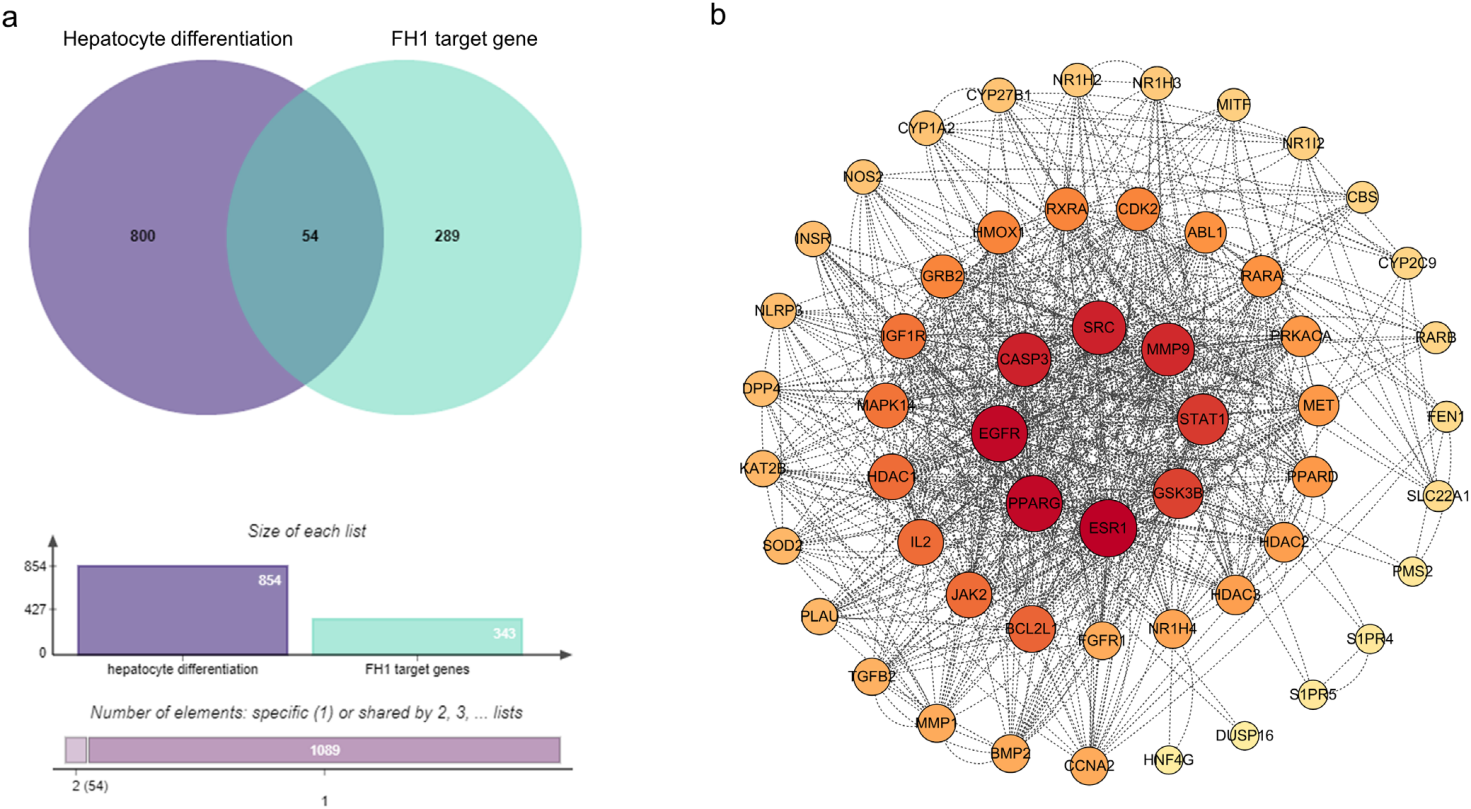
The potential target gene of FH1‐induced hepatocyte differentiation. (a) Venn diagram of the intersecting targets of FH1 and hepatocyte differentiation‐related genes. (b) PPI interaction network of FH1 targets and the intersecting targets of hepatocyte differentiation.

Searching the PharmMapper database yielded 343 targets, and searching the SEA database yielded 115 FH1 targets. After union, 343 different FH1 predicted targets were obtained. The GeneCards database was screened for targets with scores above 10, and 685 potential targets related to liver differentiation/maturation were retrieved. The OMIM database was searched for 392 gene targets, and after union, 854 predicted targets related to liver differentiation/maturation were obtained (Figure 1a).

#### 3.1.2 Analysis of Protein–Protein Interactions

Through a Venn diagram, we obtained the intersecting genes of 54 FH1 target genes and liver differentiation/maturation‐related genes (Figure 1a). We used the STRING database to construct an interaction network of the intersecting genes, which was further displayed according to the degree value (Figure 1b). A protein node in the network with a darker colour and larger size indicated a greater correlation between the protein and other proteins. Therefore, the proteins with the highest correlation according to degree were EGFR, MMP9, MET, PPARG, SRC, ESR1, STAT1 and CASP33. These findings suggest that these proteins may be key targets of FH1‐induced hepatocyte maturation.

#### 3.1.3 GO and KEGG Enrichment Analysis

**FIGURE 2:**
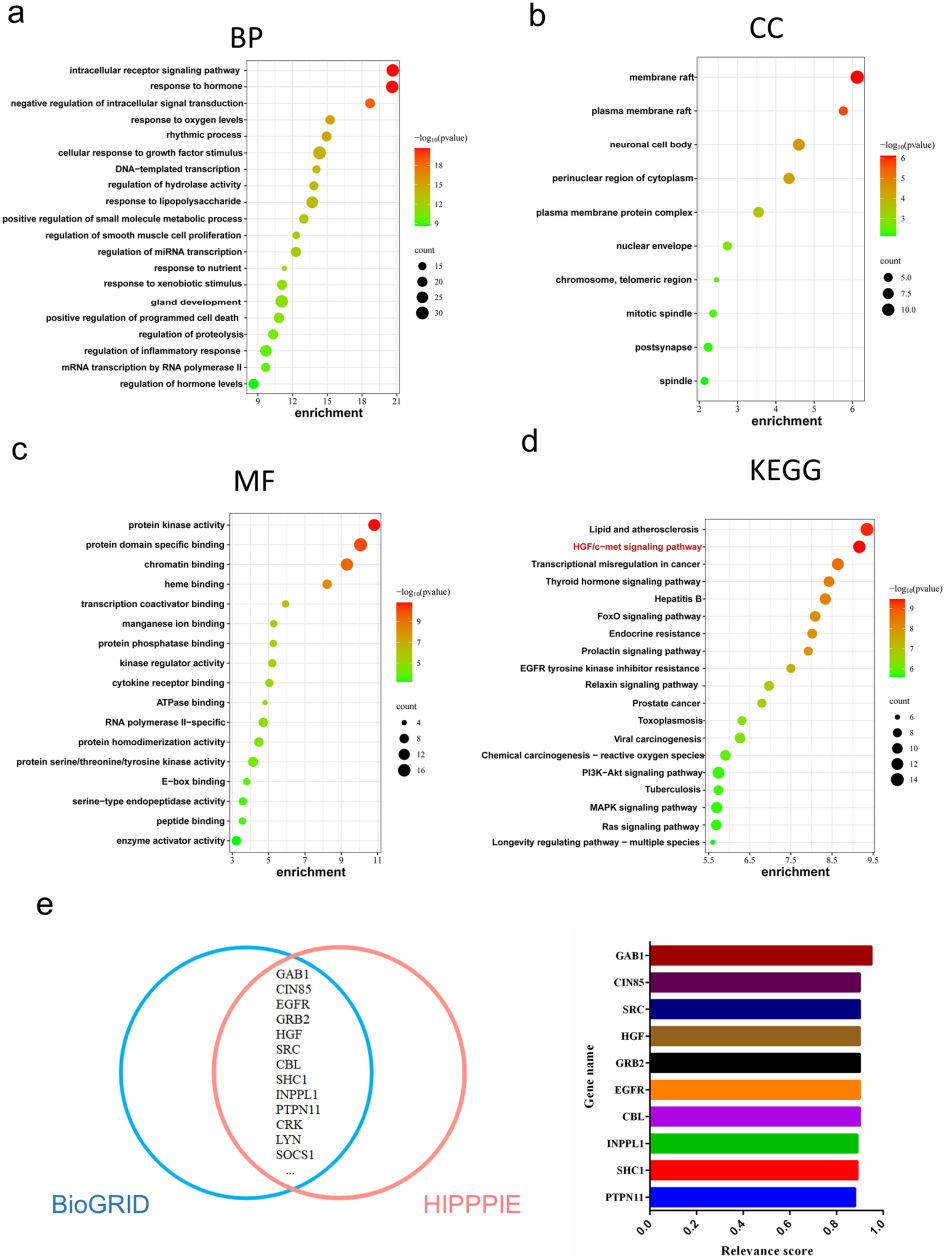
HGF/c‐Met signalling is essential for FH1‐induced hepatocyte differentiation. (a–d) GO enrichment analysis results demonstrating that the FH1‐targeted genes involved in hepatocyte differentiation are associated with biological process (BP), cellular component (CC) and molecular function (MF) categories. (e) KEGG pathway analysis results. (f) Venn diagram demonstrating the overlapping Met interactors between the BioGRID and HIPPIE databases and the HIPPIE score for overlapping proteins.

Through GO and KEGG enrichment analyses of the intersecting genes, a total of 20 GO functional entries (Figure 2a–c) and 30 KEGG signalling pathways (Figure 2d) were obtained. The cell membrane receptor signalling pathway and hormone response were found to be strongly enriched in the GO enrichment analysis. KEGG enrichment analysis revealed that the HGF/c‐Met signalling pathway was significantly correlated with FH1. We speculated that this pathway is a key FH1 pathway that regulates hepatocyte differentiation/maturation.

#### 3.1.4 Analysis of Potential Met‐Interacting Proteins

The c‐Met receptor is a disulfide‐linked heterodimeric glycoprotein that contains a kinase domain and a unique multifunctional docking site in its transmembrane β‐chain [29]. Therefore, c‐Met acts mainly through interactions with other proteins. Thus, we used BioGRID and HIPPIE (HIPPIE score > 0.7) to analyse the potential interactors of c‐Met. Thirteen proteins overlapped between the two databases. Notably, a critical regulator for inducing c‐Met internalisation and lysosomal degradation after HGF stimulation was the second most common regulatory factor according to the HIPPIE database (Figure 2e).

### 3.2 FH1 Exhibit the Potential Interaction With CIN85

**FIGURE 3:**
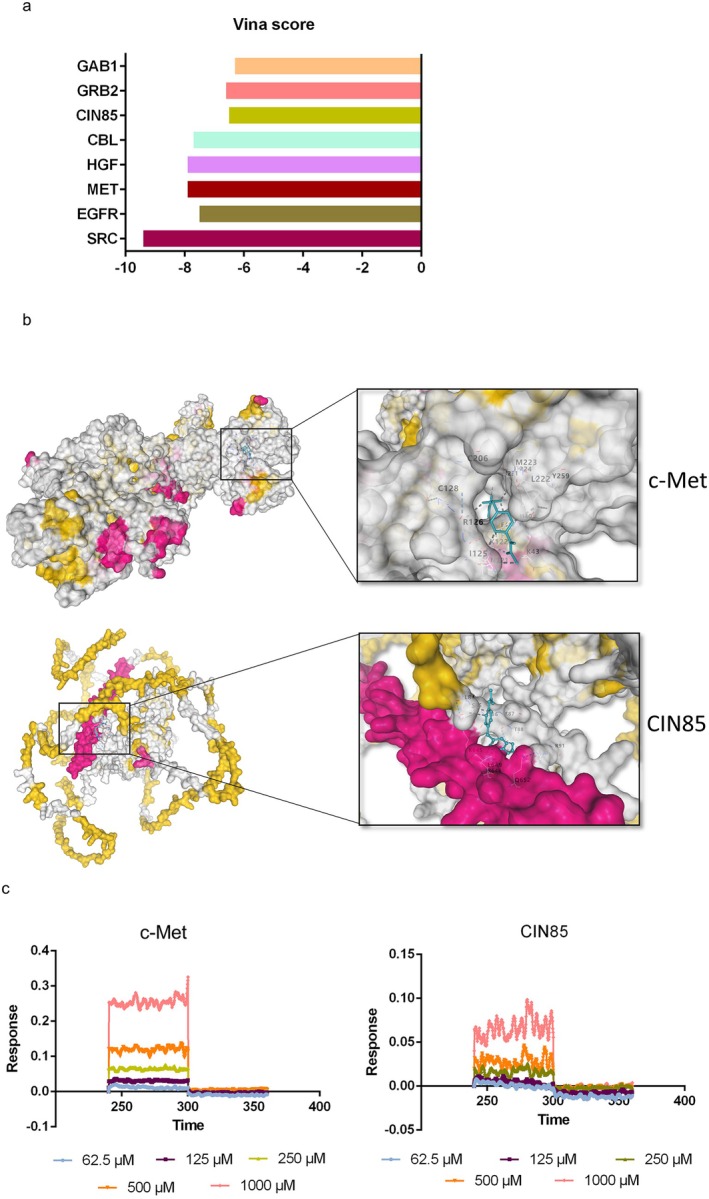
The binding of FH1 to CIN85 and c‐Met. (a) Vina score of FH1 and hub genes according to the CB. Dock 2 database. (b) Predicted binding sites of CIN85 and c‐Met for FH1. (c) Real‐time kinetic binding sensorgrams of different concentrations of FH1 (125–1000 μmol/L) are shown. The response (nm) indicates the optical thickness of the SSA biosensor layer.

Molecular docking was performed on FH1 and 7 top proteins from the BioGRID and HIPPIE databases as well as c‐Met to assess the protein‐ligand binding potential. Affinity was used to evaluate the capacity of a ligand to bind with receptors, and a lower negative affinity value indicates a stronger binding ability. Figure 3a shows the binding energy of FH1 docked with the potential proteins. Gab1 and GRB2 are vital intracellular adaptors for transmitting active c‐Met signals, with the binding energies of −6.3 and −6.6 kJ/mol with FH1 respectively. CIN85 was known as a critical regulator for inducing c‐Met internalisation and lysosomal degradation after HGF stimulation, with a binding energy of −6.5 kJ/mol with FH1. Furthermore, FH1 also had favourable docking effects with c‐Met, with a binding energy of −7.9 kJ/mol. Figure 3b illustrates the binding site and the theoretical binding mode of FH1 on CIN85 and c‐Met. In addition, we further validated the binding affinity of CIN85 and c‐Met for FH1 using BLI technology. As shown in Figure 3c, FH1 exhibited direct and reversible interactions with CIN85 and c‐Met, as revealed by the concentration‐dependent increase in response, indicating the optical thickness (nm) of the sensor layer.

### 3.3 FH1 Induce MSC Migration

**FIGURE 4:**
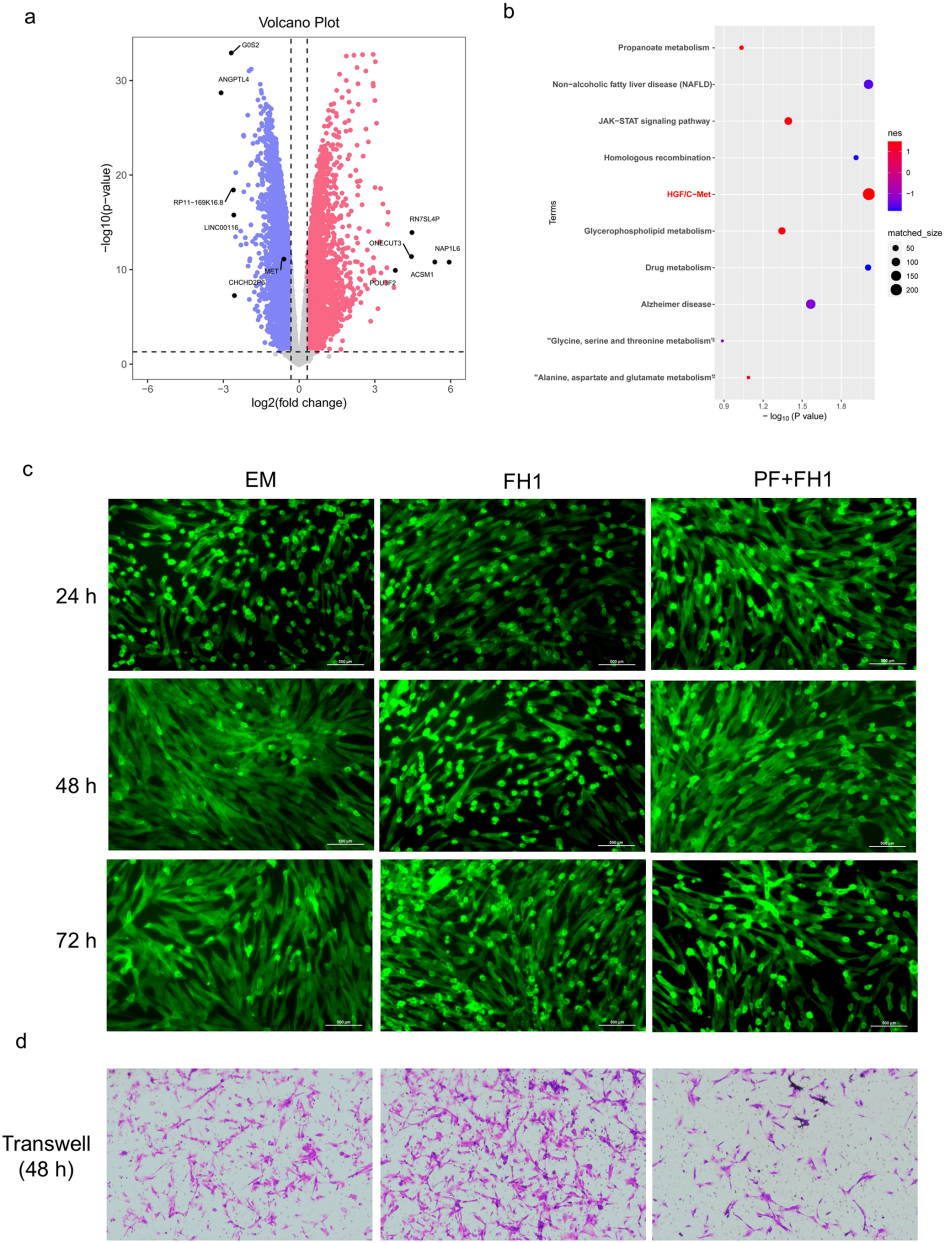
FH1 induces MSCs migration. (a) Volcano plot showing differentially expressed genes in FH1‐treated samples. (b) GSEA revealed that the differentially expressed genes were enriched mainly in the HGF/c‐Met signalling pathway. (c) MSCs seeded in six‐well plates and treated with FH1 for 24, 48 and 96 h. At the endpoint of treatment, the cells were stained with Actin‐Tracker Green‐488. The PF + FH1 group was pretreated with PF4217903 for 1 h and then cultured in medium containing 15 μM FH1 for 48 h (*n* = 3). (d) FH1 had a chemoattractant effect in hUC‐MSCs. Cells were left untreated or treated with FH1 for 48 h or pretreated with PF4217903 for 1 h before FH1 was added to the Transwell directional migration assay. All the experiments are representative of three replicates (*n* = 3).

To validate the feasibility and precision of this screening method, we first observed differences in gene expression among treated cells or untreated cells for 48 h. The RNA‐seq results demonstrated that the genes associated with differentiation were enriched in the HGF/c‐Met signalling pathway (Figure 4a,b). Then, we further investigated the effects of FH1 on MSC proliferation. We first observed changes in cell morphology after FH1 treatment for 24, 48, or 72 h through cytoskeletal staining. We found that FH1 had no obvious effect on cell morphology. Moreover, the cells still exhibited a long spindle shape after pretreatment with the c‐Met inhibitor PF4217903 for 1 h (Figure 4c). The chemoattractant effect of FH1 on MSCs was also examined through a Transwell migration assay. In the absence of c‐Met, MSCs showed a limited ability to cross the filter, whereas with FH1 (15 μM) in the lower compartment, the number of cells crossing the filter significantly increased (Figure 4d). This effect was completely inhibited by concomitant incubation with the c‐Met inhibitor PF4217903 at a concentration of 30 μM.

### 3.4 FH1 Maintains Cell Proliferation

**FIGURE 5:**
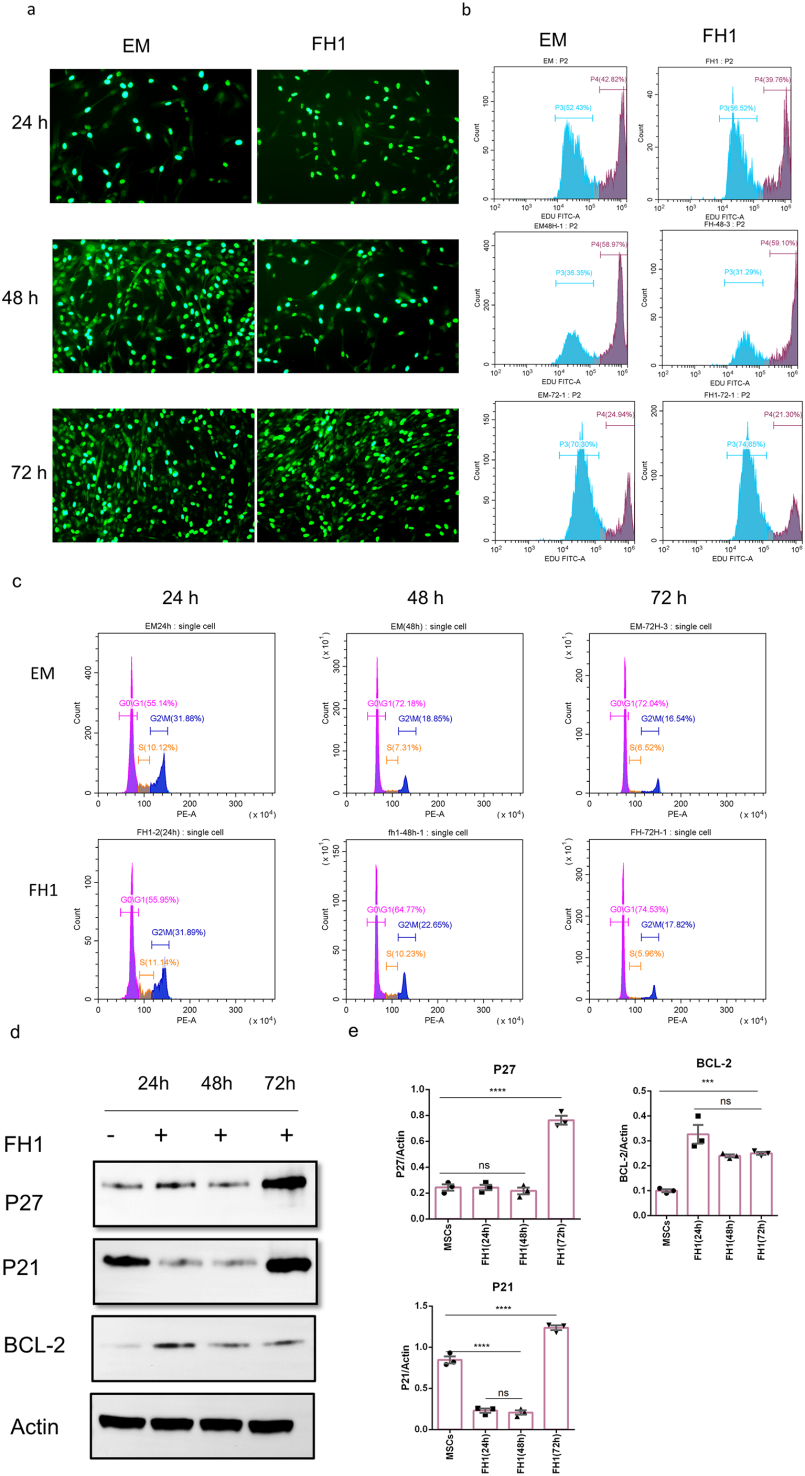
Effect of FH1 on MSCs proliferation. (a, b) Cells were seeded at a density of 5000 cells/cm^2^ in 6‐well microplates in EM and treated with or without 15 μM FH1 for the indicated times. The cells were pulsed with EdU for 2 h and then observed via fluorescence microscopy or harvested for flow cytometric analysis (*n* = 3). (c) Cells were cultured in EM with or without 15 μM FH1 treatment for 24, 48, or 72 h and then stained with 1× iodide staining solution containing 0.05 mg/mL propidium iodide, 1 mg/mL RNase A and 0.3% Triton X‐100. The cells were incubated in the dark for 30 min. The number of cells in G0/G1 phase was analysed through flow cytometry (*n* = 3). (d, e) Western blotting analysis of the protein expression levels of p21, p27 and bcl‐2 in MSCs cultured in EM medium either left untreated or treated with FH1 for the indicated times (*n* = 3). Full‐length blots/gels are presented in Figure S1. Data information: The bars represent the means of three independent experiments ± SEM. Statistics: Two‐tailed unpaired Student's *t*‐test. ***p < 0.001; ****p < 0.0001.

Small molecules can regulate stem cell fate by affecting stem cell regulatory pathways or cell cycle regulation, or preventing apoptosis [30]. In the present study, we investigated the effect of FH1 on MSC proliferation. Cells were plated at a suitable density in expansion medium (EM) and treated with 15 μM FH1 for 24, 48, or 72 h or left untreated, and cell proliferation was monitored by EdU uptake over a time course. The results showed that, compared with EM, FH1 did not induce a proliferative response in MSCs (Figure 5a,b). In a parallel experiment, cells were stained with PI, which demonstrated that FH1also did not significantly increase the G0/G1 ratio compared with that in EM without FH1 treatment. Finally, Western blot analysis revealed that FH1 can reduce or maintain the expression of the cell cycle inhibitory proteins p21 and p27 in MSCs within 48 h. Moreover, FH1 treatment induced the expression of the antiapoptotic protein bcl‐2, which is consistent with the effect of FH1 on stem cells (Figure 5c,d, Figure S1).

### 3.5 FH1 Induced the Expression of c‐Met in MSCs

**FIGURE 6:**
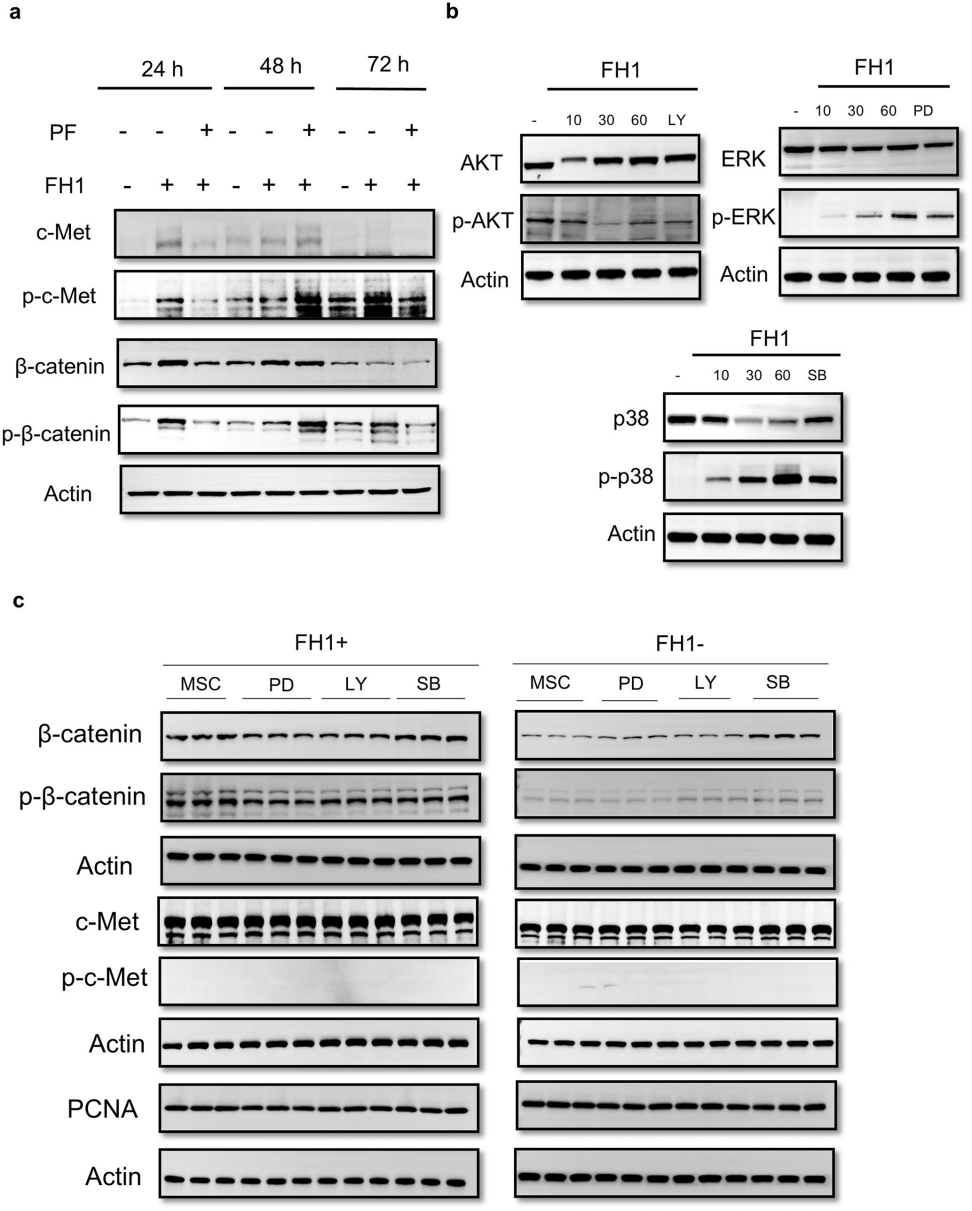
Effect of FH1 treatment on the expression of Met in MSCs. (a) Western immunoblotting analysis of c‐Met expression and FH1‐induced activation in MSCs (*n* = 3). Full‐length blots/gels are presented in Figures S2 and S3. (b) Quiescent MSCs were either left unstimulated (−) or stimulated with 15 μM FH1 for the indicated times, and total cell protein was examined through Western blotting and immunoprobed with antibodies against the active phosphorylated forms of ERK1/2, p38 and Akt (pERK1/2, pp38 and pAkt) or against total proteins (ERK1/2, p38 and Akt). Protein phosphorylation induced by 10 min of FH1 stimulation was inhibited by pretreatment for 1 h with the following specific inhibitors: PD98059 (30 M PD), SB230580 (30 M SB) and LY29402 (100 nM WM). All the experiments are representative of three replicates (*n* = 3). Full‐length blots/gels of AKT and p‐AKT are presented in Figure S4; Full‐length blots/gels of ERK and p‐ERK are presented in Figure S5; Full‐length blots/gels of p38 and p‐p38 are presented in Figure S6. (c) Western blotting analysis of the expression of Met and β‐catenin in MSCs with FH1‐induced activation. Cells treated with FH1 tend to express high levels of the receptors of Met and β‐catenin. Pretreatment with the specific inhibitors PD98059 (30 M PD), SB230580 (30 M SB), or LY29402 (100 nM WM) had no effect on the expression of Met, β‐catenin or the proliferation marker PCNA in MSCs with or without with FH1. Full‐length blots/gels are presented in Figures S7 and S8. All the experiments are representative of three replicates (*n* = 3).

According to Neuss and colleagues, human MSCs can express c‐Met [31]. To assess c‐Met expression in hUC‐MSCs, we performed Western blotting on protein extracts from MSCs. MSCs expressed low levels of Met, which was upregulated after 24 h of FH1 treatment (15 μM) and inhibited by pretreatment with the tyrosine kinase inhibitor PF4217903 for 1 h before culture in EM containing FH1. However, no phosphorylated‐c‐Met expression was identified in any of the treatment groups. In parallel, the canonical receptor of Wnt signalling was also tested, and β‐catenin was found to be expressed at low levels in MSCs but at higher levels after stimulation with FH1 for 24 h. Simultaneous treatment with PF4217903 nearly downregulated the expression of β‐catenin. Similarly, the phosphorylation of β‐catenin showed the same trend as the expression of β‐catenin in MSCs treated with FH1 (Figure 6a, Figures S2 and S3).

Because ERK1/2, p38 MAPK and PI3K are the main downstream effectors activated by HGF in liver‐derived MLP29 cell lines and MSCs [22, 32], we further investigated the potential involvement of these molecules in FH1‐stimulated MSCs. In fact, AKT phosphorylation was time independent, peaking at 10 min after FH1 treatment, whereas the phosphorylation of ERK and p38 was prolonged for 60 min. This response was eliminated by 1 h of pretreatment with specific inhibitors of ERK1/2, p38 and PI3K, namely, 30 μM PD98059, 30 μM SB230580 and 100 nM LY294002, respectively (Figure 6b, Figures [Link], [Link]). We also investigated which signalling pathway was responsible for the FH1‐dependent inhibition of cell differentiation and proliferation. As shown in Figure 6c, FH1 promoted the expression of c‐Met and β‐catenin, as well as the phosphorylation of β‐catenin. However, pretreatment with PD98059, SB230580, or LY294002 for 1 h had no effect on the phosphorylation of c‐Met in MSCs treated with or without FH1. Moreover, as shown by analysis of the expression of PCNA, the proliferation of MSCs did not change (Figure 6c, Figures S7 and S8).

### 3.6 FH1‐iHeps Exhibited the Phenotypic Traits of Hepatic Progenitor Cells

**FIGURE 7:**
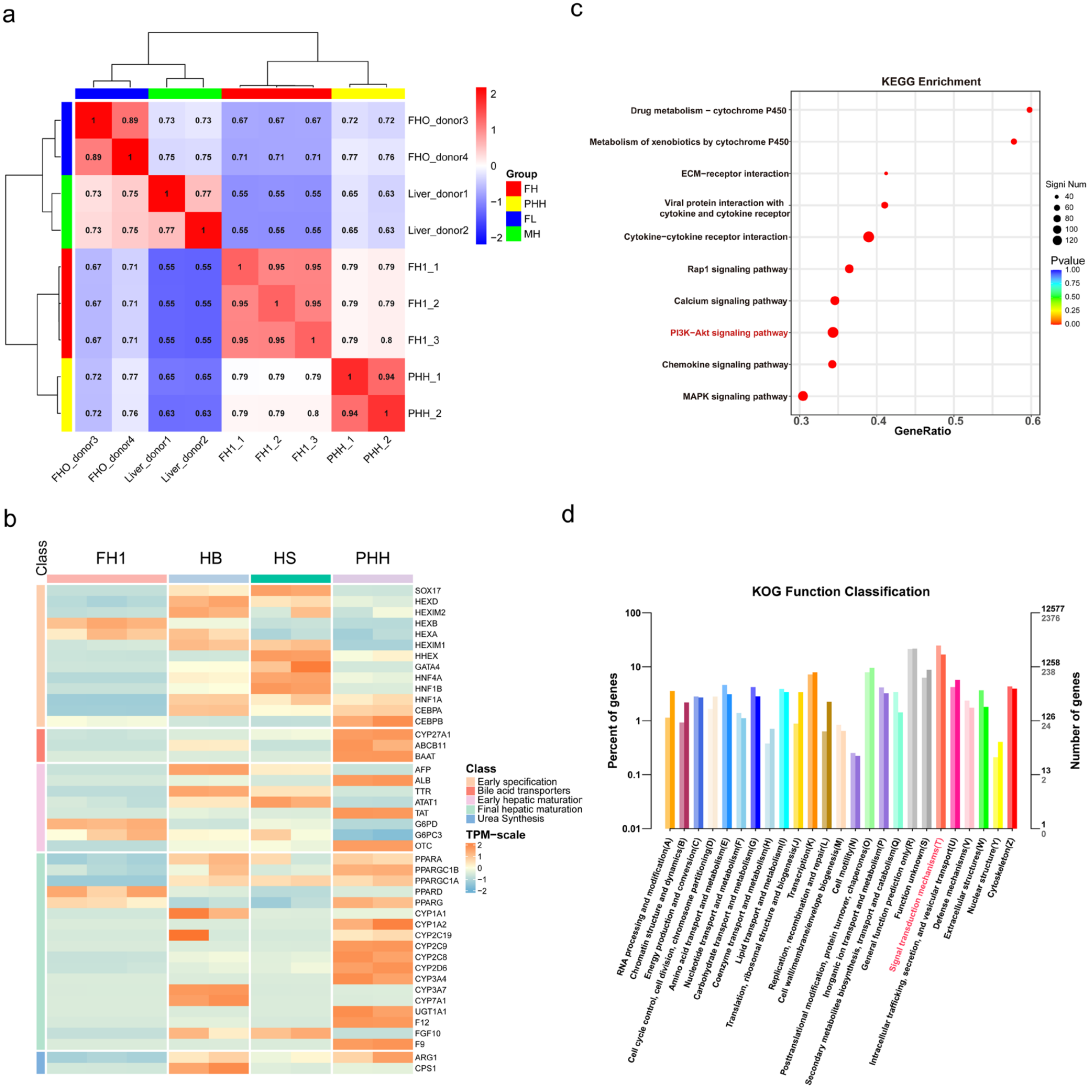
The RNA profile of FH1‐induced hepatocytes. (a) Hierarchical clustering of PHHs, FH1‐iHeps, HSs and HBs according to differentially expressed genes. (b) Heatmap showing the expression of selected genes in PHHs versus FH1‐iHeps. (c) KEGG pathway analysis results. (d) KOG results showing the differential signalling pathway‐related functions.

The degree of similarity between FH1‐induced HLCs and primary hepatocytes was analysed. We analysed the gene expression profile of FH1‐iHeps using RNA sequencing and examined primary hepatocytes (GEO: GSM3683651, GSM3683652), FL (GEO: GSM6598973, GSM6598974) and MH (GEO: GSM6598965, GSM6598966) in a public database. We identified a correlation between FH1‐iHeps and PHHs is 0.79 (Figure 7a), but these two cell types still had gene expression differences. Gene expression profiling revealed that most of the genes associated with hepatocyte maturation were expressed at significantly lower levels than in primary hepatocytes (Figure 7b). However, it is worth noting that the significant upregulation of hepatic progenitor cell marker genes DLK and TROP2 suggests that the efficiency of differentiation of hUC‐MSCs toward the mature stage of hepatocytes might be less than optimal and the cellular differentiation was still stagnant at the stage of hepatic progenitor cell formation. KEGG and GO analysis showed that the differentially expressed genes between the two cell types were mainly enriched in the PI3K‐AKT signalling pathway, which is a downstream signalling pathway of HGF/c‐Met and is closely related to liver development. Thus, although FH1 could induce the directed differentiation of MSCs into HLCs, these cells still differed from PHHs in terms of cellular composition (Figure 7c,d).

### 3.7 Therapeutic Potential of FH1‐iHeps in CCl_4_‐Induced ALF

**FIGURE 8:**
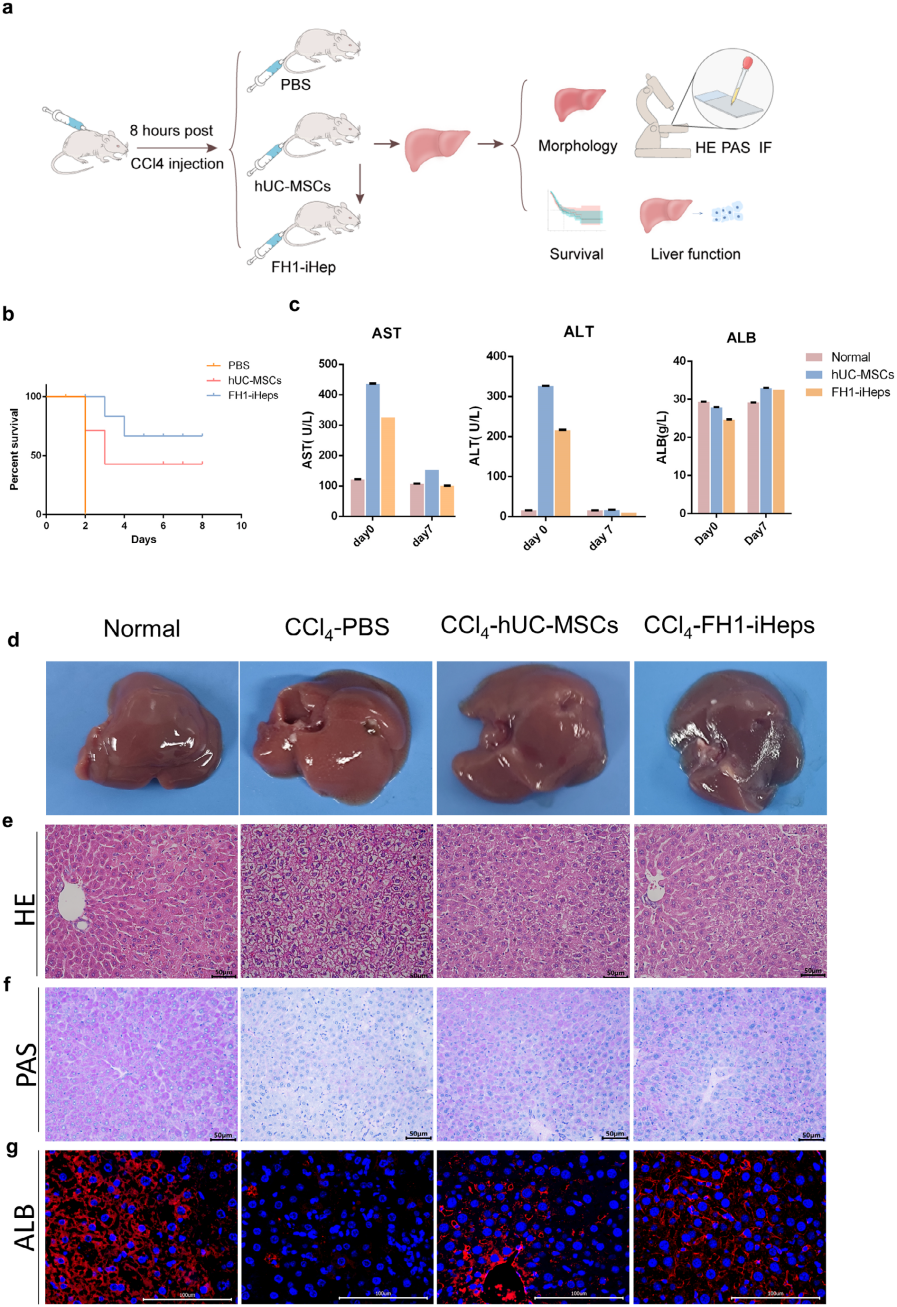
The therapeutic effects of FH1‐ iHeps on ALF. (a) Schematic diagram of cell transplantation into the livers of C57BL/6J mice. Mice were intraperitoneally injected with CCl_4_ to induce fulminant hepatic injury. Eight hours after CCl_4_ treatment, hUC‐MSCs and iHeps (1 × 10^6^ cells/animal, 300 μL) were intravenously injected into the mice, and the control animals received an equal volume of PBS. (b) Kaplan–Meier survival curve of mice with ALF (*n* = 6). (c) Serum levels of ALT, AST and human albumin (ALB) in CCl_4_‐treated mice before (day 0 [d0]) and after (d7) transplantation of Huc‐MSCs or iHeps (*n* = 3). (d) Macroscopic images of freshly isolated livers from mice treated with CCl_4_ for 8 h; PBS, hADSCs and iHeps were intravenously injected into the mice (*n* = 3). (e) H&E staining of liver sections (*n* = 3). (f) PAS staining (*n* = 3). (g) The integration of iHeps in mouse livers was determined by immunostaining for human ALB in serial sections (*n* = 3). Scale bars = 50 μm (e, f) and 100 μm (g). Data information: The bars represent the means of three independent experiments ± SEM. Statistics: Two‐tailed unpaired Student's *t*‐test.

To further determine whether FH1‐iHeps could exert liver function in vivo, we observed the in vivo reparative effects of FH1‐iHeps through mouse transplantation experiments. C57BL/6J mice were injected with CCl_4_ to trigger ALF. Eight hours after transplantation, the mice were randomly divided into three groups: the control group was intravenously injected with 300 μL of PBS, the hUC‐MSC group was injected with 300 μL of cell suspension containing 1 × 10^6^ hUC‐MSCs, and the FH1‐iHeps group was injected with 300 μL of cell suspension containing 1 × 10^6^ FH1‐iHeps (Figure 8a). The animals in the control group died within 24 h, and the survival rates of the mice transplanted with hUC‐MSCs and FH1‐iHeps were 30% and 60%, respectively (Figure 8b). Transaminase activity experiments revealed significantly upregulated expression of serum glutamic‐pyruvic transaminase (ALT) and glutamic oxalacetic transaminase (AST) in the transplantation groups, which had approximately normal levels (Figure 8b–d). Pathological analysis revealed that FH1‐iHeps improved the recovery from CCl_4_‐induced liver damage (Figure 8e–g). The surviving mice that received FH1‐iHeps treatment showed repopulation of the liver parenchyma. Taken together, these findings indicate that FH1‐iHeps may be a potential clinical treatment for acute liver injury.

## 4 Discussion

**FIGURE 9:**
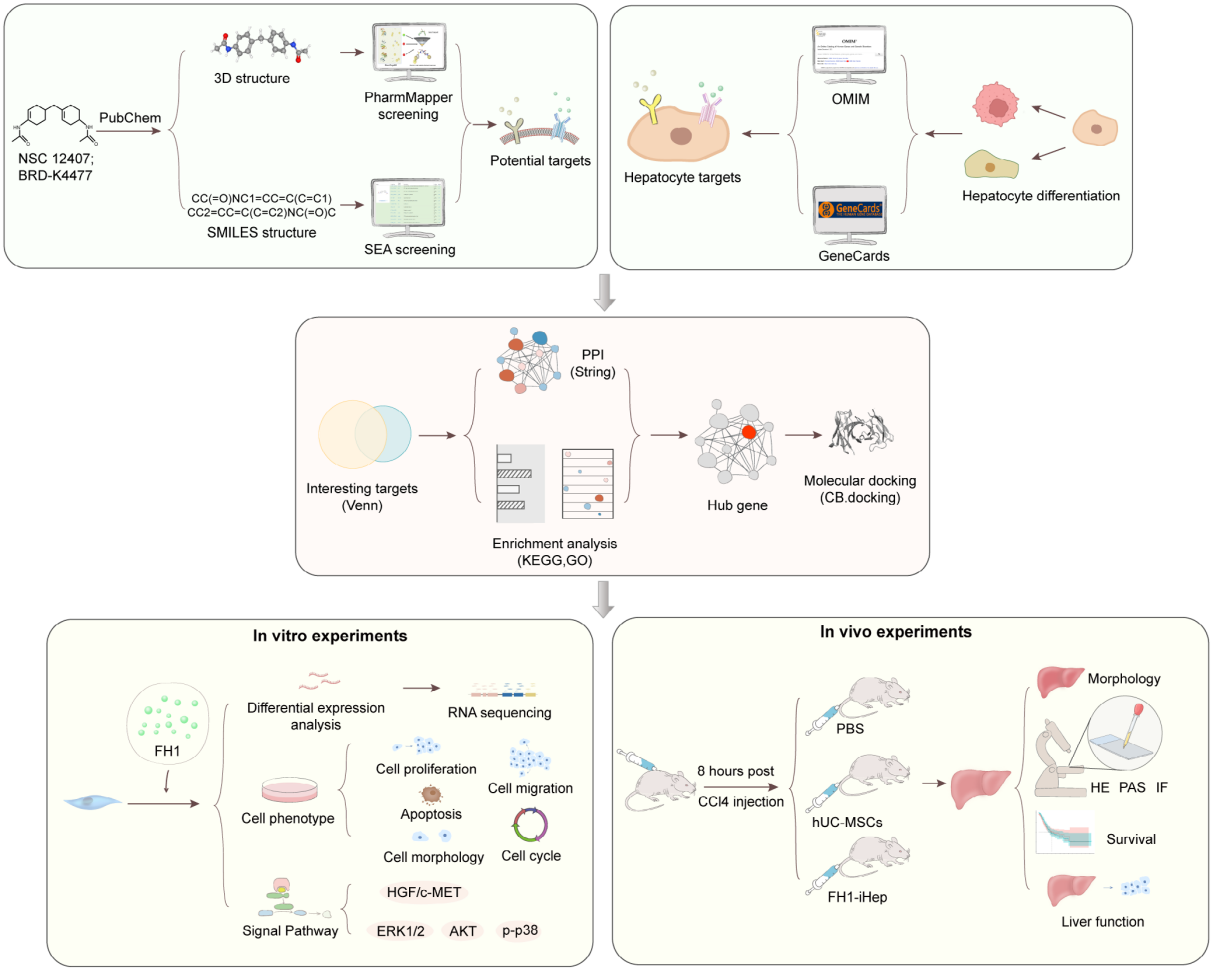
The flowchart of this study.

ALF is a serious human health risk, with approximately 2 million people dying from ALF each year, accounting for 3.5% of all deaths worldwide [33]. Hepatocellular dysfunction is the main cause of worsening ALF; however, due to the lack of liver donors, timely treatment is not provided for many patients, resulting in the loss of life. Primary hepatocytes provide a new strategy for the treatment of ALF, but the number of primary hepatocytes is limited, and long‐term culture in vitro tends to result in loss of function; therefore, the search for high‐quality and sufficient hepatocytes for the treatment of ALF is still a major obstacle in the clinic [34, 35]. The strategy of using hepatocytes to treat liver failure depends on the simple hypothesis that liver function can be improved by the use of exogenous hepatocytes. MSCs have many functions, such as immunoregulation, multi‐differentiation and repair of damaged tissue. Many previous studies have shown that rodent‐ and human‐derived MSCs can differentiate into different species. Human‐derived MSCs can differentiate into HLCs both in vitro and in vivo, especially driven by FH1‐based strategy established in our previous work [36, 37, 38, 39]. Therefore, elucidating the mechanism by which FH1 regulates stem cells to directionally differentiate into hepatocytes is valuable for the establishment of a new model of hepatocytes (Figure 9).

In 2016, Zhang et al. [30] reported that small molecule compounds induce changes in stem cell fate mainly by regulating stem cell signalling pathways, cell cycle progression and apoptosis; therefore, the effects of small molecule compounds on the biological functions of stem cells are a prerequisite for determining their ability to differentiate along an established lineage. In this study, we investigate the effects of FH1 on the proliferative capacity of MSCs, the alteration of the cell cycle G0/G1 cell ratio, and the differences in the expression levels of the cell cycle inhibitory proteins P21 and P27 and the antiapoptotic molecule bcl‐2. Surprisingly, FH1 maintains cell proliferation in the G0–G1 phase, which is a mechanism mediated by altered expression levels of the p21 and p27 proteins, which are known as universal cell cycle progression inhibitors that act by binding to cyclin‐CDK complexes and PCNA [40, 41]. However, FH1 significantly increased the expression of antiapoptotic molecules. The arrest of cell proliferation was dependent on p38 MAPK because it was abrogated by treatment with its specific inhibitor, SB203580. p38 has also been shown to play a similar role in FGF‐dependent chondrocyte proliferation [42]. We also observed the chemotactic effect of FH1 on MSCs, but its recruitment to extrahepatic regions deserves specific analysis in future studies.

At the stage of hepatocyte maturation, Met is a strong inducer of hepatocyte differentiation [43]; therefore, whether FH1 induces hepatocyte maturation through the activation of this signalling attracted our interest. With respect to the characteristics of FH1, network pharmacology is a novel and well‐documented method in clinical research [44]. Network pharmacology has been applied to explore the basic pharmacological effects and mechanisms of Chinese herbal preparations [45]. In this study, we predicted the key target of FH1 in liver development and maturation and the related signalling pathway, the HGF/c‐Met signalling pathway, by network pharmacology analysis, and the enriched genes were found to be highly associated genes. Since Met is a key target molecule of this signalling pathway, we obtained key protein targets with potential interactions with Met through the BioGRID database (https://thebiogrid.org) and the HIPPIE database (http://cbdm‐01.zdv.unimainz.de/~mschaefer/hippie/index.php). Through molecular docking experiments and biostratigraphic interference techniques, FH1 was found to have an affinity of −6.5 kJ/mol for the Met signalling regulatory molecule CIN85 and −7.9 kJ/mol for Met. Our results revealed that FH1 induces cellular‐directed hepatocyte differentiation mainly through the HGF/c‐Met signalling pathway.

In a previous study, HGF/Met signalling was shown to facilitate progenitor and embryonic stem cell differentiation and induce pluripotent stem cell differentiation into a hepatocyte lineage [46, 47, 48]. However, whether FH1 similarly regulates HGF/c‐Met in MSCs, thereby inducing their differentiation into liver lineage cells, has not been reported. After analysing the differences in the transcript levels of MSCs before and after 48 h of FH1 treatment by RNA‐seq, we found that the differentially expressed genes were enriched mainly in the HGF/c‐Met signalling pathway, a finding that validated the results of the network pharmacology analysis. Subsequently, we observed changes in cell morphology and migration following the addition of FH1 and the c‐Met inhibitor PF4217903 during MSC culture, and the results showed that FH1 did not significantly induce morphological changes in MSCs; however, cell migration was significantly reduced after treatment with the c‐Met inhibitor PF4217903. Since stem cell development is regulated by the Wnt signalling pathway [49], we analysed the intracellular levels of activation/phosphorylation of c‐Met, a ligand of the HGF signalling pathway and β‐catenin, a ligand of the Wnt signalling pathway, in the presence of FH1 and a c‐Met inhibitor by Western blots. The results revealed that the MSCs without FH1 expressed low levels of the proteins β‐catenin and c‐Met. In fact, the phosphorylation level of intracellular β‐catenin was significantly increased after FH1 treatment for 48 h; however, the phosphorylation of c‐Met in MSCs could not be induced regardless of the presence or absence of FH1 and c‐Met inhibitors.

The Met receptor was reported to be a delayed early gene whose expression in epithelial cells can be upregulated by treatment with serum, phorbol esters, or HGF itself [50]. After full ligand‐dependent tyrosine phosphorylation, the Met receptor expressed on MSCs can activate the Ras‐ERK1/2 and p38 MAPK pathways as well as the PI3K/Akt pathway, which are the main transduction pathways activated in other HGF‐responsive cells within a few minutes [51]. Depending on the cell type, these pathways were reported to variably contribute to the different biological responses elicited by HGF. The Ras‐ERK1/2 MAPK pathway was shown to be mostly associated with HGF mitogenic and morphogenic effects, whereas the PI3K/Akt pathway is mainly related to HGF mitogenic and morphogenic effects. Another pathway seems to be related mainly to motogenic and antiapoptotic effects [44]. In our study, we found that the expression levels of PCNA, an indicator of cell proliferation, were not significantly altered in the presence or absence of FH1 or in the presence of AKT, ERK, or p38 inhibitors; these findings differ from those in HGF‐interacting cells.

Mimicking developmental biology in an in vitro system has, and still does, required several revisions and refinements. Hepatic maturation is regularly monitored by the selective up‐ and down‐regulation of a plethora of genes. Unfortunately, RNA‐seq analysis revealed that FH1derived HLC differed significantly from PHH at the gene level. This was particularly evident in aspects related to the functions of mature hepatocytes, such as Phase I and II activities, bile acid transporters, and enzymes involved in the urea cycle, which suggests that the maturation efficiency of FH1 on MSC is limited.

Finally, we observed the reparative effects of iHeps in mice following transplantation into CCl_4_‐induced mice. The repair effect of the FH1‐iHeps on the livers of mice with ALF as well as the mice treated with MSCs alone was noted. Previous studies showed that tissue regeneration based on MSC treatment is not mainly related to cell differentiation [52], but is highly correlated with a variety of secreted factors for example, HGF, SCF [53]; GLPx3 [54]; FGF21 [55], mitochondrial translocation [56] and exocytosis [57]. Several groups suggest that fusion events that occurred between donor and host hepatocytes may be the key mechanism by which cell transplantation exerts its reparative effects [58, 59, 60, 61]. Whether FH1‐iHeps fuses with host liver tissue and functions is worthy of further study.

Although the discovery of this mechanism of action can provide a research basis for hepatocyte differentiation, MSC‐derived HLC obtained by either method still faces problems of cryogenic preservation and limited yield after enzymatic isolation and long‐term survival. Maybe we, through mapping the lineage‐specific progression of cells from endodermal precursors to fetal HB and postnatal hepatocytes, need to refine the existing induction strategies to improve the current limitations of human stem cell‐derived HLC, especially in terms of safety and efficacy criteria for clinical translation or in vitro drug toxicity assessment. On the other hand, this study has not yet investigated longer survival, changes in liver function and pathological structures such as fibrosis levels and tissue inflammatory infiltration after transplantation of MSC and FH1‐iHeps, and perhaps the treatment that may be needed after undergoing long‐term cell transplantation, such as in patients with chronic liver failure, may differ.

## 5 Conclusions

In summary, we identified the key signalling pathway involved in the FH1‐induced hepatocyte differentiation. FH1‐iheps have the potential to treat ALF, even if those cells do not fully acquire the function of mature hepatocytes. In the present study, we also found that FH1 interacts with key signalling molecules of c‐Met, but whether FH1 induces hepatocyte differentiation by affecting the CIN85‐c‐Met interaction deserves further exploration.

## Author Contributions

**Sang Luo:** funding acquisition (equal), writing – original draft (equal), writing – review and editing (equal). **Fang Wu:** data curation (equal), methodology (equal), software (equal). **Yiran Jin:** methodology (equal). **Dan Liu:** investigation (equal), project administration (equal), writing – review and editing (equal).

## Ethics Statement

We have strictly adhered to the ethical principles concerning animal studies. This study was reviewed and approved by the Ethics Committee of General Hospital of Ningxia Medical University (1) Title of the approved project: Biological Function of Liver organoids derived from human mesenchymal stem/stromal cells. (2) Name of the institutional approval committee: Ethics Committee of General hospital of Ningxia Medical University. (3) Approval number: KYLL‐2023‐0169. (4) Date of approval: 7 November 2023.

## Consent

The authors have nothing to report.

## Conflicts of Interest

The authors declare no conflicts of interest.

## Supporting information


Figure S1.

Figure S2.

Figure S3.

Figure S4.

Figure S5.

Figure S6.

Figure S7.

Figure S8.

## Data Availability

The data supporting this study's findings are available from the corresponding author upon reasonable request. Bulk RNA‐seq data of FH1‐treated hUC‐MSCs were provided in Sequence Read Archive (No. PRJNA1138884, https://www.ncbi.nlm.nih.gov/sra/PRJNA1138884). Bulk RNA‐seq data of FH1‐derived induced hepatocyte‐like cells were provided in Sequence Read Archive (No. PRJNA1139665, https://www.ncbi.nlm.nih.gov/sra/PRJNA1139665).
