# Supplementary figures and images for: The Potential Hepatocyte Differentiation Targets and MSC Proliferation by FH1
Source: J Cell Mol Med. 2025 May 10;29(9):e70601. doi: 10.1111/jcmm.70601 (PMC12064995; doi:10.1111/jcmm.70601)

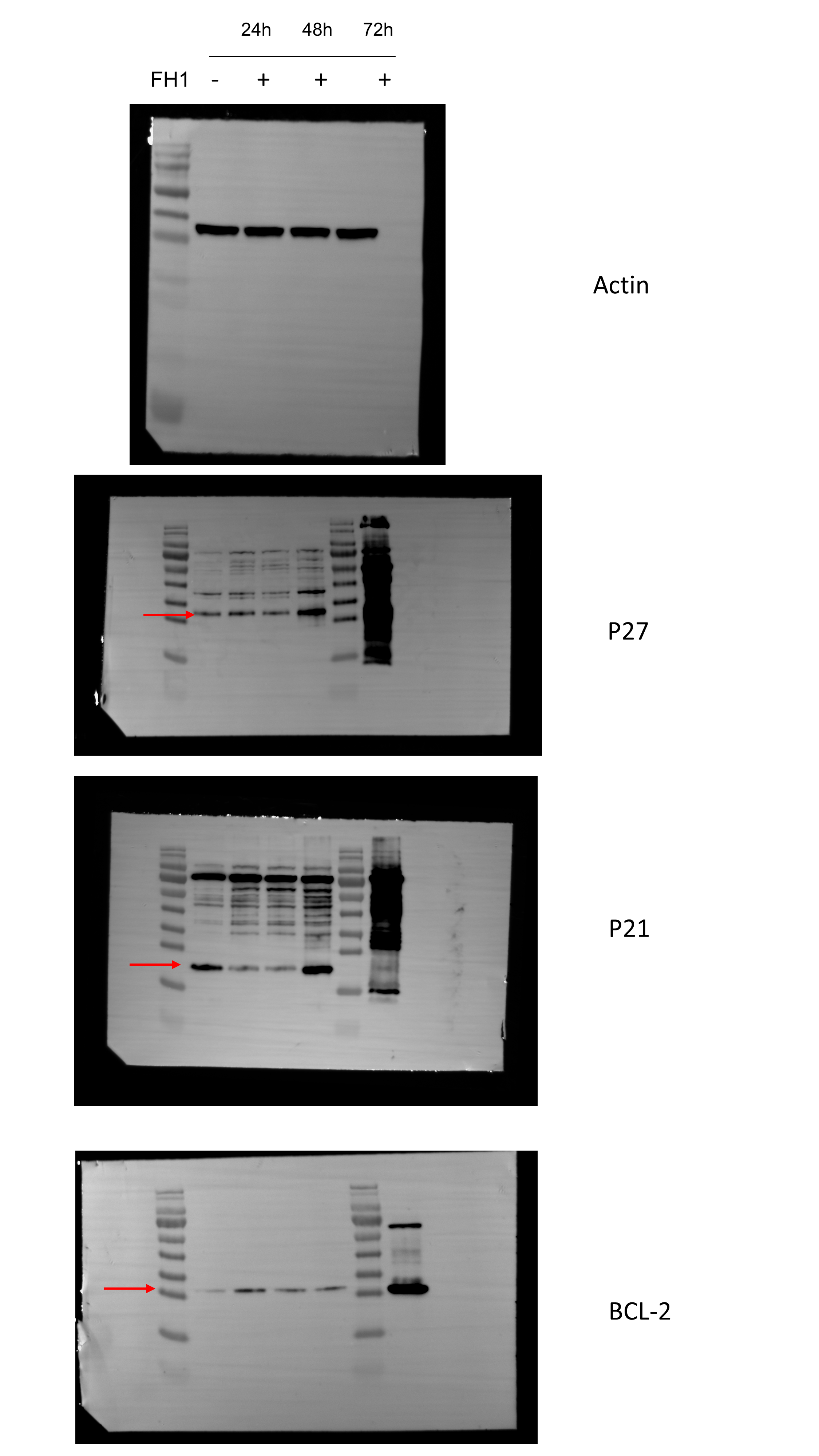

Supplement: Supplementary file 1 — Figure S1. [file JCMM-29-e70601-s001.tif]

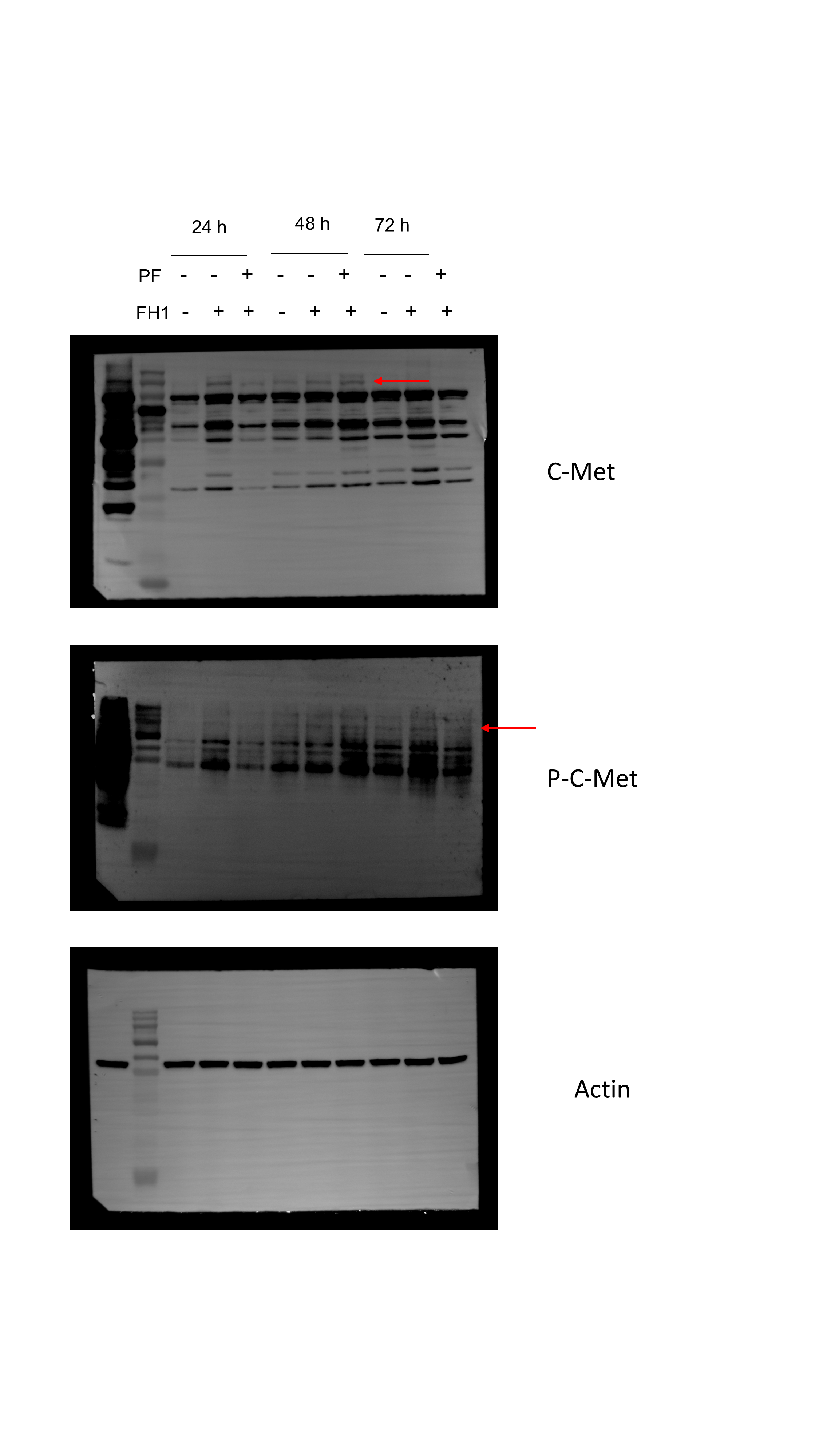

Supplement: Supplementary file 2 — Figure S2. [file JCMM-29-e70601-s002.tif]

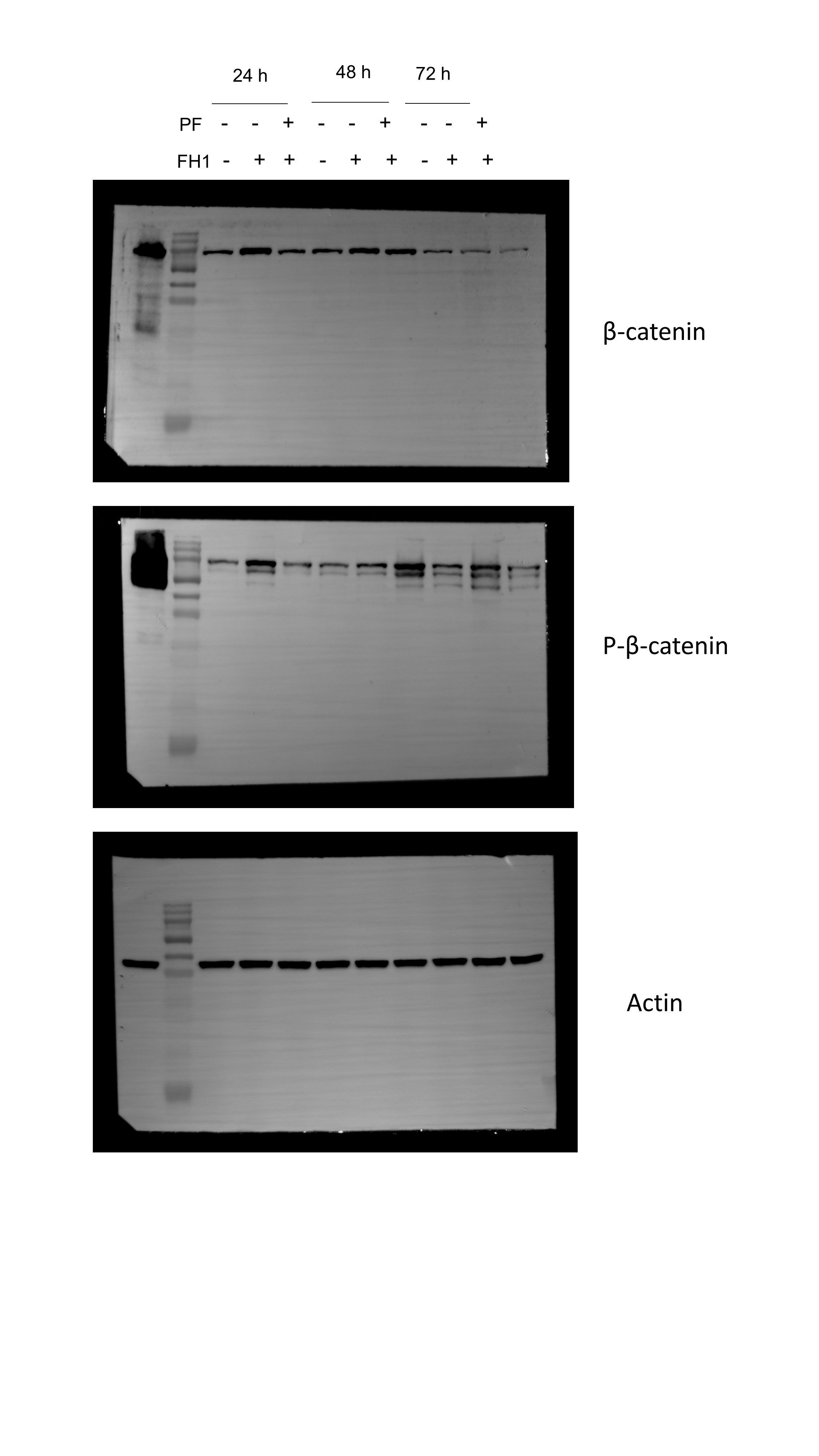

Supplement: Supplementary file 3 — Figure S3. [file JCMM-29-e70601-s003.tif]

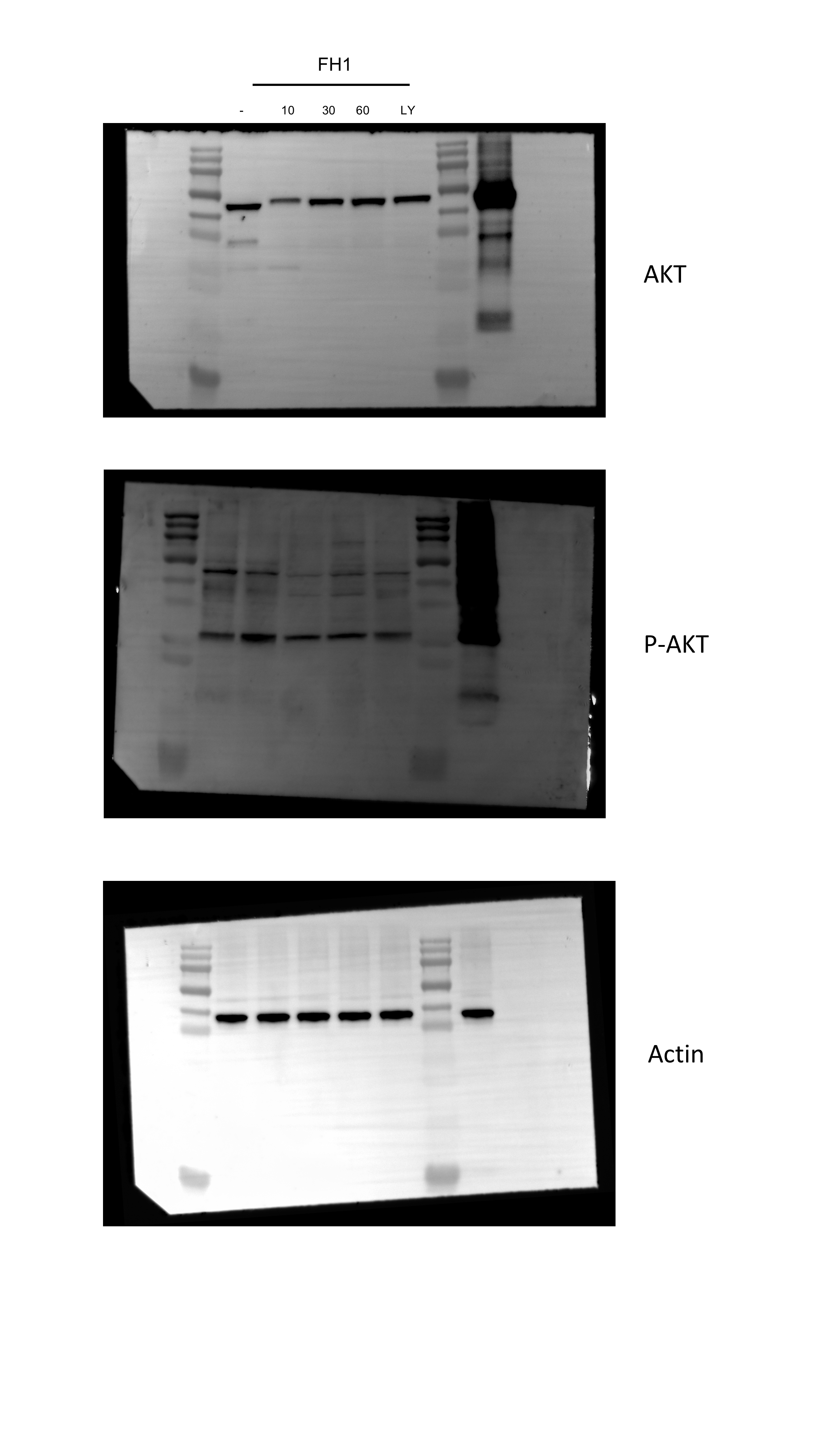

Supplement: Supplementary file 4 — Figure S4. [file JCMM-29-e70601-s005.tif]

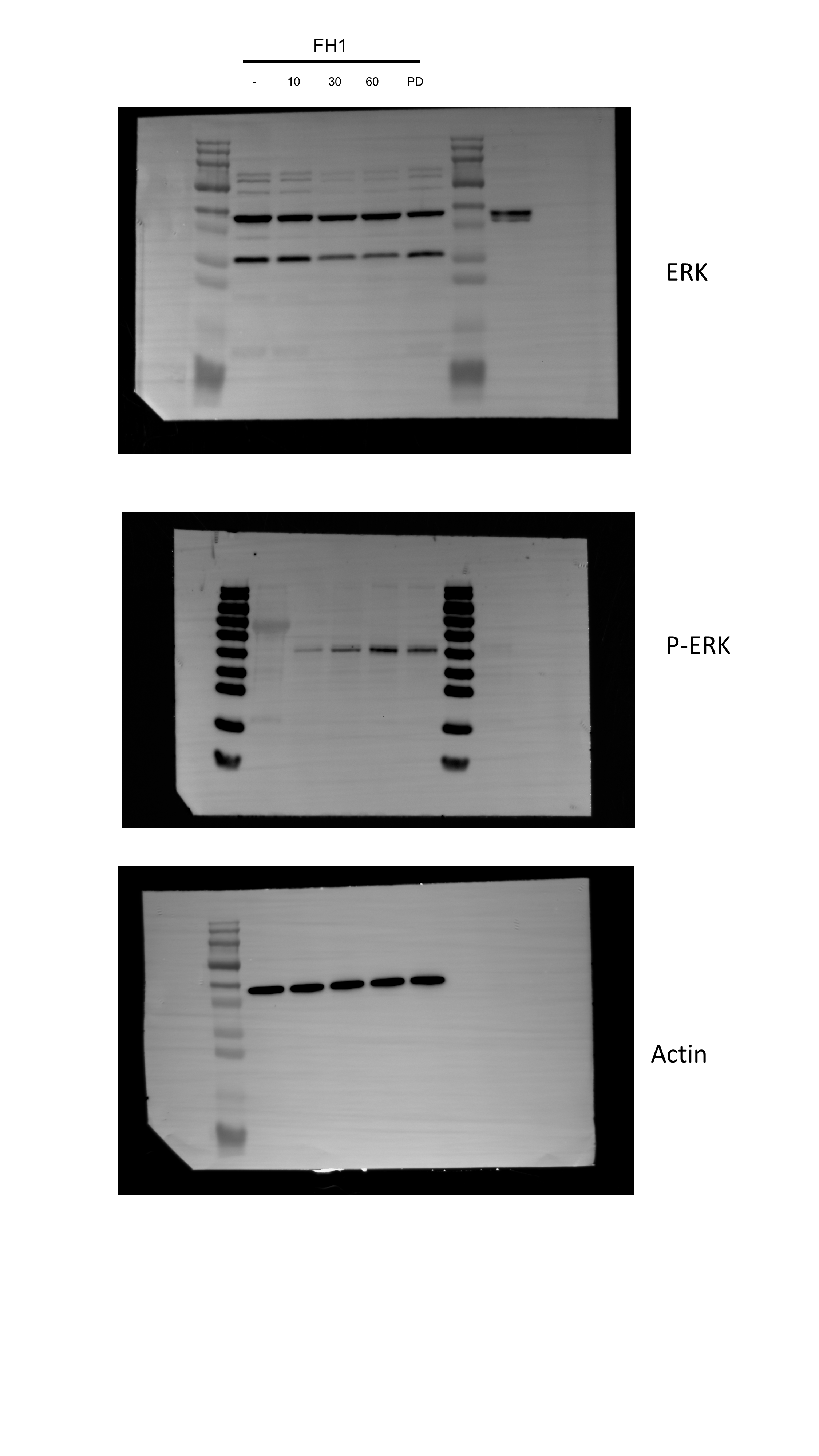

Supplement: Supplementary file 5 — Figure S5. [file JCMM-29-e70601-s004.tif]

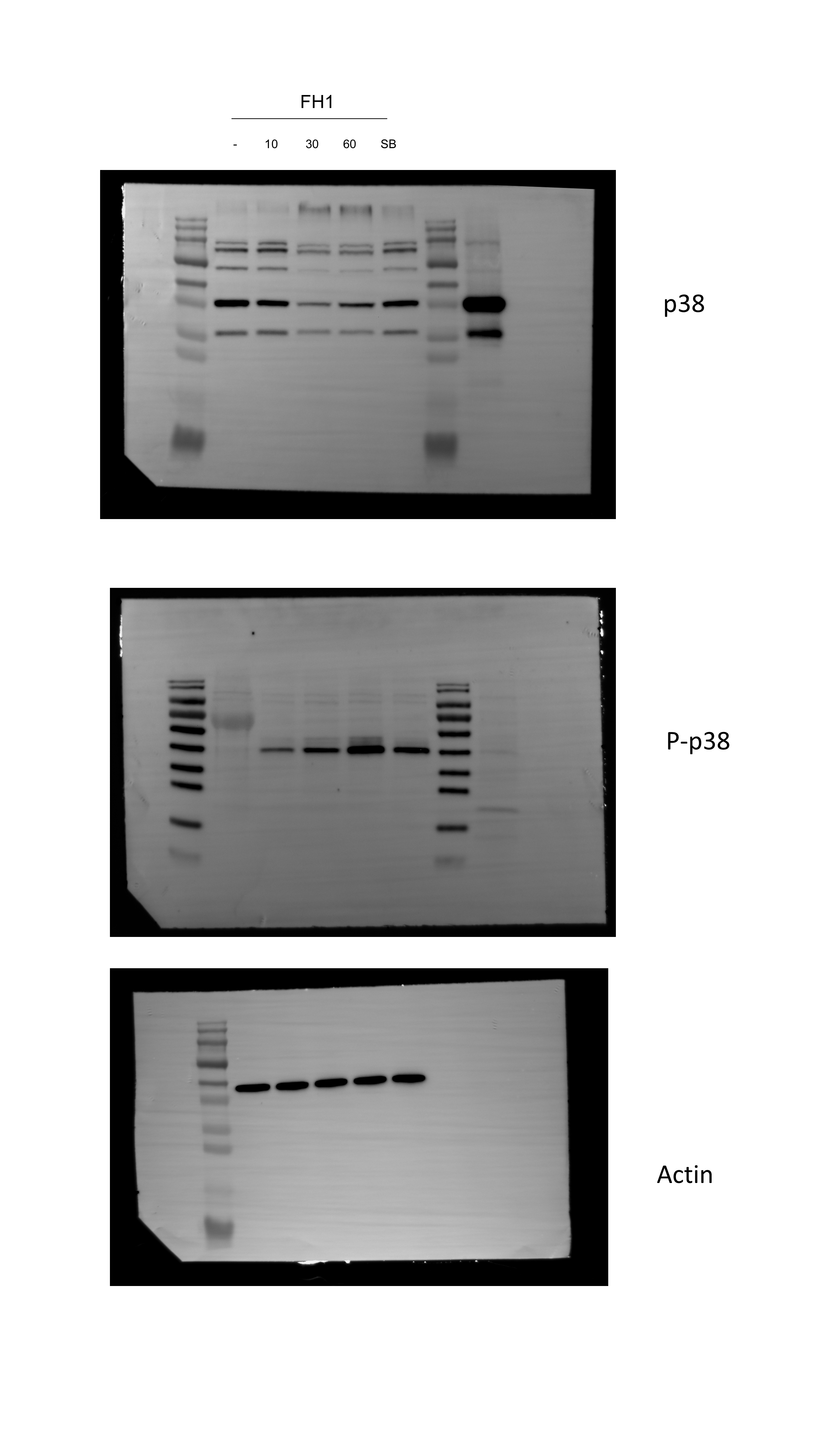

Supplement: Supplementary file 6 — Figure S6. [file JCMM-29-e70601-s008.tif]

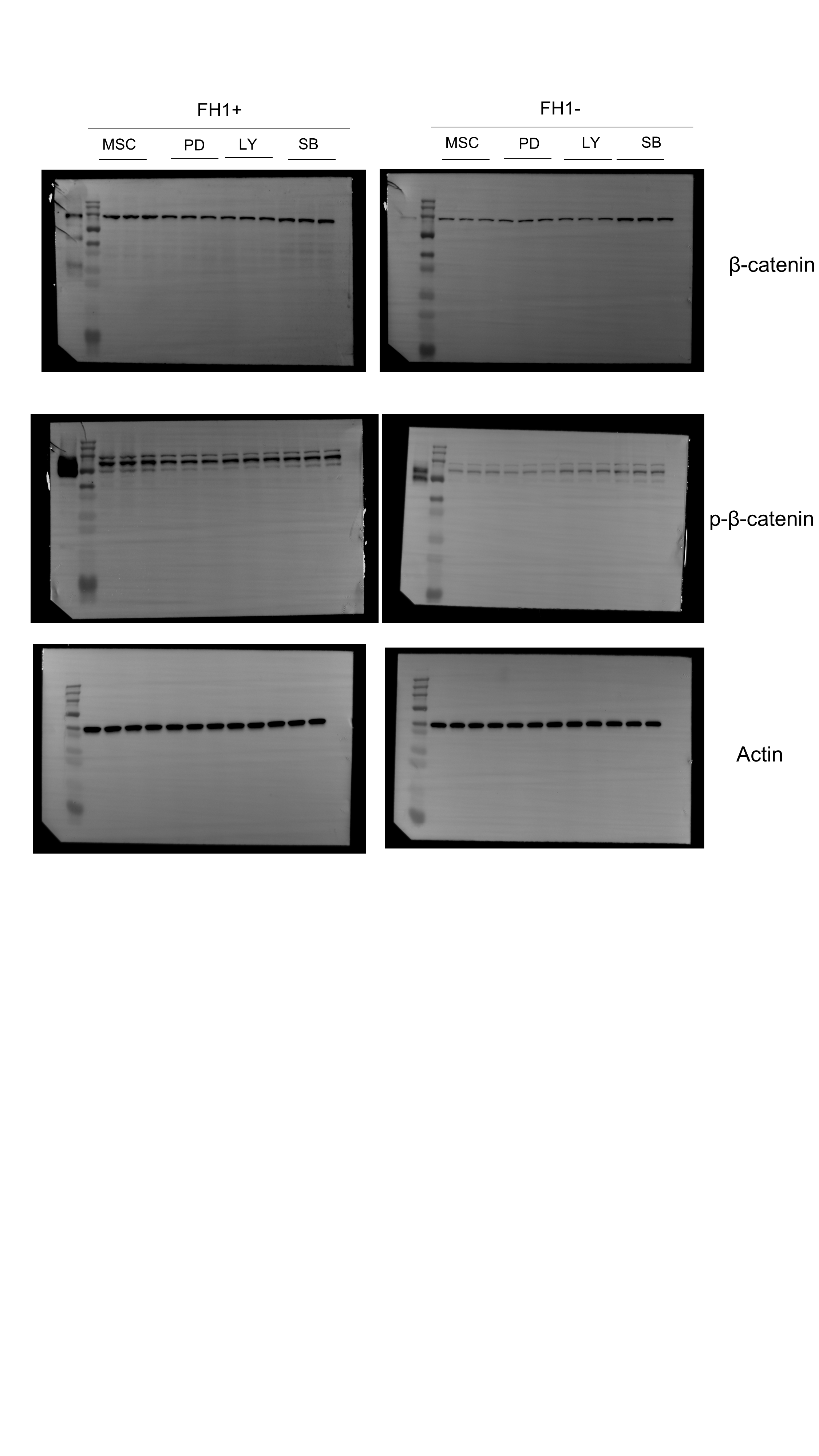

Supplement: Supplementary file 7 — Figure S7. [file JCMM-29-e70601-s007.tif]

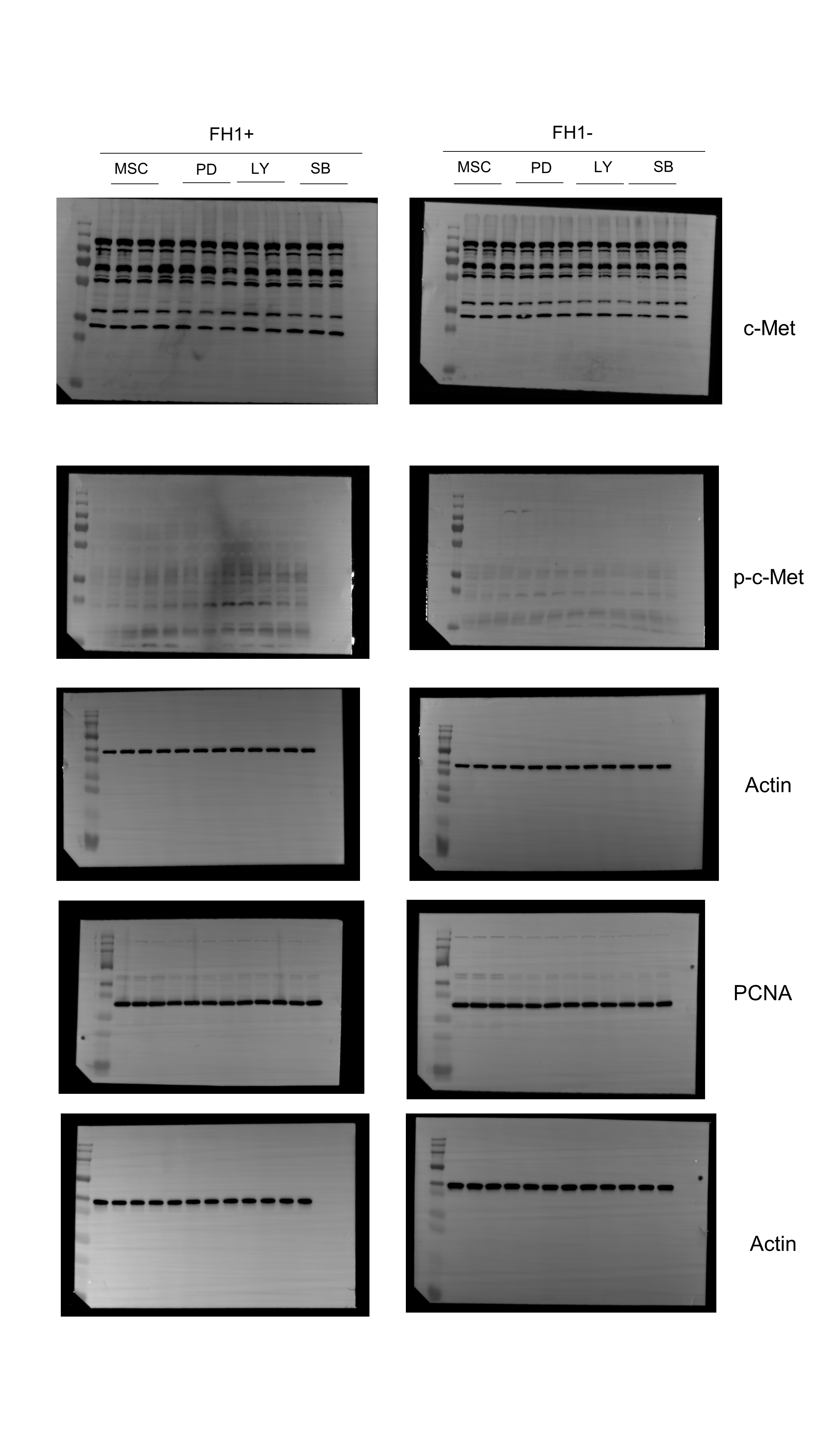

Supplement: Supplementary file 8 — Figure S8. [file JCMM-29-e70601-s006.tif]
